# A phase 2, double-blind, multicenter, randomized, placebo-controlled, dose‑ranging study of the efficacy and safety of Astodrimer Gel for the treatment of bacterial vaginosis

**DOI:** 10.1371/journal.pone.0232394

**Published:** 2020-05-04

**Authors:** Arthur S. Waldbaum, Jane R. Schwebke, Jeremy R. A. Paull, Clare F. Price, Stephanie R. Edmondson, Alex Castellarnau, Philip McCloud, George R. Kinghorn

**Affiliations:** 1 Downtown Women’s Health Care, Denver, CO, United States of America; 2 Division of Infectious Diseases, University of Alabama at Birmingham, Birmingham, AL, United States of America; 3 Starpharma Pty Ltd, Melbourne, VIC, Australia; 4 McCloud Consulting Group, Sydney, NSW, Australia; 5 Royal Hallamshire and Sheffield Teaching Hospitals, Sheffield, United Kingdom; GERMANY

## Abstract

**Background:**

Astodrimer Gel contains a novel dendrimer intended to treat and prevent bacterial vaginosis. We assessed the efficacy and safety of Astodrimer Gel for treatment of bacterial vaginosis.

**Methods:**

132 women with bacterial vaginosis were randomized 1:1:1:1 to Astodrimer 0.5% (N = 34), 1% (N = 33), or 3% (N = 32) Gel or hydroxyethyl cellulose placebo gel (N = 33) at a dose of 5 g vaginally once daily for 7 days at 6 centers in the United States. The primary endpoint was clinical cure (no bacterial vaginosis vaginal discharge and no more than one of 1) vaginal pH ≥4.5; 2) ≥20% clue cells; or 3) positive whiff test) at study days 21–30. Secondary analyses included clinical cure at study days 9–12, patient-reported symptoms, acceptability and adverse events.

**Results:**

The Astodrimer 1% Gel dose was superior to placebo for the primary and selected secondary efficacy measures in the modified intent-to-treat population. Clinical cure rates at day 9–12 were superior to placebo for the Astodrimer 3%, 1% and 0.5% Gel groups (62.5% [15/24; *P* = .002], 74.1% [20/27; *P* < .001], and 55.2% [16/29; *P* = .001], respectively, vs. 22.2% [6/27]). At day 21–30, clinical cure rates were 46.2% (12/26) for the 1% dose vs. 11.5% for placebo (3/26; *P* = .006). A greater proportion of patients reported absence of vaginal discharge and vaginal odor at day 9–12 and day 21–30 for Astodrimer Gel groups compared with placebo. Adverse events considered potentially treatment-related occurred in only 25% of Astodrimer Gel-treated patients vs. 22% of placebo patients.

**Conclusion:**

Astodrimer Gel once daily for 7 days was superior to placebo for treatment of bacterial vaginosis and was well-tolerated. The 1% dose consistently showed the strongest efficacy across endpoints. These results support a role for Astodrimer Gel, 1%, as an effective treatment for bacterial vaginosis.

## Introduction

Bacterial vaginosis (BV) is the most common vaginal infection worldwide and is approximately twice as common as vulvovaginal candidiasis [1]. BV is associated with serious health consequences, including pre-term birth, and acquisition and transmission of human immunodeficiency virus (HIV) and other sexually transmitted infections (STIs) [2] [3].

Most common treatments for BV are oral or vaginal antimicrobial drugs [4] [5]. Antibiotics, including various 5-nitroimidazole derivatives, metronidazole, tinidazole and secnidazole, and clindamycin are associated with high rates of candidiasis when used for treatment of BV [6] and are not well-tolerated by patients due to gastrointestinal side effects or interaction with alcohol. Current treatment options for BV are inadequate and alternate therapies are required [7] [8] [9].

Astodrimer is of the class of novel compounds called dendrimers, characterized by a highly branched, three-dimensional architecture. Astodrimer is a generation-four lysine dendrimer with a polyanionic surface charge [10]. Both size and surface charge of the dendrimer contribute to the function of the compound [11]. Astodrimer is formulated in an aqueous, Carbopol^®^-based, muco-adhesive gel. Astodrimer has been shown to inhibit growth of bacteria associated with BV via a novel mechanism of action compared with conventional antibiotics, by blocking the attachment of bacteria to cells and inhibiting the formation of and disrupting biofilms. Bacterial biofilms are important in the pathogenesis of BV and are not adequately targeted by existing therapies, leading to inadequate treatment and frequent recurrence [9] [12] [13] [14]. Astodrimer is well-tolerated and not systemically absorbed [15] [16].

Therefore, Astodrimer Gel offers the potential to be a non-antibiotic alternative that avoids issues associated with antibiotics. The current study assessed different doses of Astodrimer Gel for treatment of women with BV.

## Materials and methods

### Study design

This was a double-blind, multicenter, randomized, placebo-controlled, dose-ranging study assessing the efficacy and safety of three strengths (0.5%, 1%, and 3%) of Astodrimer Gel applied vaginally for 7 days compared with placebo (hydroxyethyl cellulose placebo gel) [17] [18] in women with BV.

The study complied with the Declaration of Helsinki, was conducted in accordance with Good Clinical Practice, regulatory guidelines, and relevant local legislation, and was approved by an institutional review board on July 23, 2010 (Quorum Review, Inc.). Patient enrolment commenced August 23, 2010 and last follow up was on February 25, 2011. The study was registered on clinicaltrials.gov on 12 September 2010 as NCT01201057. Registration was delayed with respect to first patient enrolled in line with US regulatory standards, which required registration within 21 days of the first patient being enrolled. The authors confirm that all ongoing and related trials for this product are registered.

All patients provided written informed consent and were screened for eligibility at the baseline visit (study day 1). Eligible patients were randomized in a 1:1:1:1 ratio to receive 0.5%, 1% or 3% Astodrimer Gel or placebo gel using a computer-generated randomization list based upon a permutation block procedure. The three active gels and placebo were colorless gels packaged in identical, pre-filled, vaginal applicators. Each applicator contained a single-dose (5 g) and was individually overwrapped in a sealed pouch. Eight overwrapped applicators (7 doses and 1 spare) were packed in a tamper-evident carton labeled with a unique patient identification number (PIN). Each patient was assigned a PIN according to the randomization schedule and the corresponding carton was dispensed at baseline. Women self-administered a dose vaginally once a day for 7 days and then attended an end of treatment (EOT) visit between study days 9-12 for response assessments and evaluation of adverse events (AEs). A 2-3 week follow-up period concluded with a test of cure (TOC) visit between study days 21-30. Both care providers and patients were unaware of treatment allocations throughout the study. Patients could withdraw from the study at any time.

Women who reported no improvement in BV symptoms or who relapsed could request rescue therapy, provided the investigator confirmed the presence of BV. Women who received rescue medication were considered failures for clinical cure but were assessed for safety up to the final study visit.

The 3 gel strengths were chosen based on microbiology data. The 0.5% strength was chosen as data indicated efficacy against *Gardnerella vaginalis* without affecting *Lactobacillus* species (spp). The 3% strength was chosen as it provided the broadest activity against *Bacteroides ovatus*, *P*. *bivia* and *G*. *vaginalis*, and the 1% strength was selected to investigate a mid-range dose.

### Study population

Women aged 18-45 years with a diagnosis of BV, defined as presence of 4 Amsel criteria for BV (discharge; vaginal fluid pH ≥4.5; ≥20% clue cells; and positive 10% potassium hydroxide whiff test) [19] and Nugent score (NS) ≥4 [20] were randomized. The protocol allowed enrolment before confirmation of a NS ≥4 if an OSOM^®^ BVBlue^®^ test (Sekisui Diagnostics; Burlington MA, USA) returned a positive result.

Women who were pregnant, planning to become pregnant, lactating, or who were not willing to use contraception during the study, and women testing positive for urinary tract infection (UTI), or *Chlamydia trachomatis*, *Neisseria gonorrhoeae* or *Trichomonas vaginalis* infections at screening were excluded. Patients who had received antifungal or antimicrobial therapy (systemic or intravaginal) within 14 days of enrolment were excluded from the study.

Concomitant systemic and topical antimicrobial therapies and vaginal antifungal therapies were not permitted during the study, and the use of other vaginally administered products and oral antifungals was discouraged.

### Outcomes

The primary efficacy endpoint was clinical cure defined as normal physiological discharge, and meeting no more than one other of 1) vaginal pH ≥4.5; 2) ≥20% clue cells; or 3) positive whiff test at day 21–30. Clinical cure was also assessed at day 9–12, constituting a secondary efficacy endpoint. At the time of study conduct, the US Food and Drug Administration (FDA) required the assessment at study day 21–30 be the primary endpoint (FDA Guidance for Industry, titled *Bacterial Vaginosis; Developing Antimicrobial Drugs for Treatment*, issued July 22, 1998, withdrawn August 7, 2013). In 2016, FDA re-issued its guidance on BV (FDA Guidance for Industry, titled *Bacterial Vaginosis; Developing Antimicrobial Drugs for Treatment*, issued July 13, 2016), recommending the primary assessment of cure be at study day 7–14. This timing is consistent with study days 7-10 for assessment of treatment success proposed by a US National Institutes of Health (NIH) workshop on BV clinical practice [5], and with the assessment at days 9–12 used in the current study. Other secondary endpoints assessed at study days 9–12 and 21–30 were Nugent cure (NS ≤3 when a score of ≥7 was determined at baseline); therapeutic cure, a composite of clinical cure and Nugent cure; and therapeutic resolution of BV, defined as clinical cure and NS <6.

Additional efficacy measures included resolution of each individual Amsel criteria; NS; use of rescue medication; presence of BV symptoms as assessed by the investigator and the patient; acceptability of study treatment from the Treatment Satisfaction Questionnaire for Medication (TSQM, Version 1.4) at day 9–12; and semi-quantitative culture analysis of vaginal flora.

AEs were monitored throughout the study.

### Statistical analyses

A formal interim analysis was conducted after completion of the TOC visit for 50% of the participants. Data displays were produced by an independent statistician and programmer who were not part of the project team. An O’Brien-Fleming α-spending function was used for the interim analysis. No changes to the study were made on the basis of the interim analysis. The significance calculated for the final analysis was α = .048.

Primary and secondary efficacy analyses using the Cochran-Mantel-Haenszel (CMH) test controlling for study center were performed on the modified Intent-To-Treat (mITT) population, which was all patients randomized who had administered ≥1 dose of study product, NS ≥4 at baseline, a post-baseline assessment of efficacy (NS or Amsel criteria) and non-use of prohibited medications that may have confounded efficacy assessment.

The safety population was all patients randomized who had received ≥1 dose of study product. The microbiological population was a subset of the ITT population and included patients that had at least one sample collected for semi-quantitative culture analysis of vaginal microflora.

Continuous efficacy variables (e.g., questionnaires or NS) were compared between all treatment groups using analysis of variance (ANOVA) adjusting for study center. The semi-quantitative culture analysis of vaginal microflora was presented descriptively.

### Sample size calculation

Assuming clinical cure rates of 50% and 15% for Astodrimer Gel and placebo, respectively, a sample size of 28 evaluable patients per treatment arm provided 80% power to detect a treatment difference with a final alpha significance level of α = .048. Assuming a 15% attrition rate, 33 patients per treatment arm and 132 patients overall were to be randomized.

## Results

### Disposition and demographics

**Fig 1:**
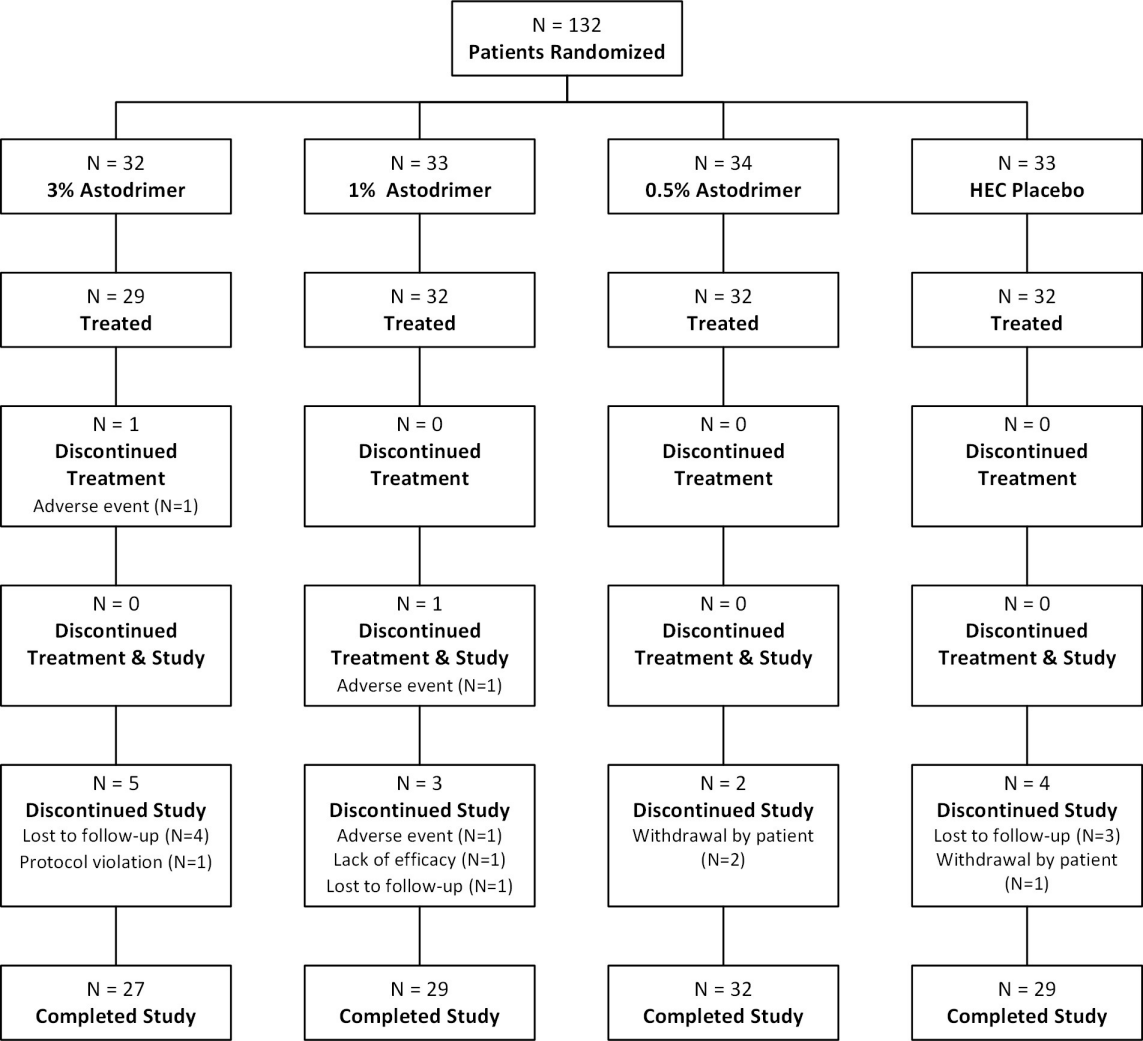
Consort diagram. Patient enrollment by treatment group.

A total of 132 eligible women were randomized to receive placebo (N = 33), astodrimer 3% (N = 32), astodrimer 1% (N = 33) or astodrimer 0.5% (N = 34) at 6 sites in the US. The mITT population included 111 women. Treatment groups were well-balanced with respect to demographic and baseline characteristics (Table 1). As presented in Fig 1, the majority of patients in all treatment groups completed treatment (at least 6 days) and the study.

**Table 1:** Baseline characteristics (mITT population), by treatment group.

	Astodrimer Gel			Placebo
	3% (N = 26)	1% (N = 27)	0.5% (N = 30)	(N = 28)
**Age (yr)**				
Mean (SD)	29.5 (7.24)	30.0 (6.66)	29.0 (7.68)	30.6 (7.66)
Range	19 to 44	18 to 42	18 to 45	18 to 44
**Race, n (%)**				
White	12 (46.2%)	17 (63.0%)	19 (63.3%)	14 (50.0%)
Black	14 (53.8%)	10 (37.0%)	9 (30.0%)	11 (39.3%)
All Others^a^	0	0	2 (6.7%)	3 (10.7%)
**Ethnicity, n (%)**				
Non-Hispanic	20 (76.9%)	20 (74.1%)	22 (73.3%)	22 (78.6%)
Hispanic	6 (23.1%)	7 (25.9%)	8 (26.7%)	6 (21.4%)
**Nugent score, n (%)**				
4 to 6	5 (19.2%)	3 (11.1%)	8 (26.7%)	3 (10.7%)
7 to 10	21 (80.8%)	24 (88.9%)	22 (73.3%)	25 (89.3%)

N = number of patients analyzed, n = number of patients with observation, mITT = modified intent-to-treat, SD = standard deviation

^a^ All Others = Asian, Native American

### Efficacy

Each of the astodrimer groups was superior to placebo for clinical cure at day 9–12 (statistically significant), with the highest cure rate in the 1% astodrimer group: 74.1% (20/27) vs. 22.2% (6/27) in placebo (*P* < .001) (Table 2). At day 21–30, the primary outcome measure of clinical cure rate for the 1% astodrimer group was statistically significantly higher than placebo: 46.2% (12/26) vs. 11.5% (3/26), respectively (*P* = .006).

**Table 2:** Efficacy outcomes (mITT population), by treatment group.

	Astodrimer Gel			Placebo
	3%	1%	0.5%	
	n/N (%)	n/N (%)	n/N (%)	n/N (%)
	[95% CI]	[95% CI]	[95% CI]	[95% CI]
	*P*-value^a^	*P*-value^a^	*P*-value^a^	
**Clinical Cure**				
** Day 9–12**	15/24 (62.5)	20/27 (74.1)	16/29 (55.2)	6/27 (22.2)
	[40.6, 81.2]	[53.7, 88.9]	[35.7, 73.6]	[8.6, 42.3]
	*P* = .002	*P* < .001	*P* = .010	
** Day 21–30**	7/25 (28.0)	12/26 (46.2)	7/30 (23.3)	3/26 (11.5)
	[12.1, 49.4]	[26.6, 66.6]	[9.9, 42.3]	[2.4, 30.2]
	*P* = .158	*P* = .006	*P* = .248	
**Nugent Cure**				
** Day 9–12**	6/25 (24.0)	5/26 (19.2)	6/29 (20.7)	0/27 (0)
	[9.4, 45.1]	[6.6, 39.4]	[8.0, 39.7]	[0.0, 12.8]
	*P* = .006	*P* = .025	*P* = .015	
** Day 21–30**	3/25 (12.0)	5/26 (19.2)	3/30 (10.0)	1/26 (3.8)
	[2.5, 31.2]	[6.6, 39.4]	[2.1, 26.5]	[0.1, 19.6]
	*P* = .361	*P* = .069	*P* = .412	
**Therapeutic Cure**				
** Day 9–12**	2/24 (8.3)	7/26 (26.9)	5/29 (17.2)	2/27 (7.4)
	[1.0, 27.0]	[11.6, 47.8]	[5.8, 35.8]	[0.9, 24.3]
	*P* > .500	*P* = .073	*P* = .219	
** Day 21–30**	4/25 (16.0)	5/26 (19.2)	3/30 (10.0)	2/26 (7.7)
	[4.5, 36.1]	[6.6, 39.4]	[2.1, 26.5]	[0.9, 25.1]
	*P* = .493	*P* = .247	*P* > .500	
**Therapeutic Resolution**				
** Day 9–12**	13/24 (54.2)	13/26 (50.0)	11/29 (37.9)	3/27 (11.1)
	[32.8, 74.4]	[29.9, 70.1]	[20.7, 57.7]	[2.4, 29.2]
	*P* = .001	*P* = .006	*P* = .018	
** Day 21–30**	5/25 (20.0)	9/26 (34.6)	3/30 (10.0)	3/26 (11.5)
	[6.8, 40.7]	[17.2, 55.7]	[2.1, 26.5]	[2.4, 30.2]
	*P* = .491	*P* = .062	*P* > .500	

CI = confidence interval, N = number of patients analyzed, n = number of patients with observation

^a^ Pairwise *P*-value comparing each astodrimer gel strength to placebo

Similarly, a greater proportion of patients in each of the astodrimer groups compared with placebo met the criteria for Nugent cure at day 9–12 (statistically significant for each group) and day 21–30 (*P* = .069 for 1% astodrimer group).

The clinical and Nugent cure results were supported by therapeutic cure and therapeutic resolution data, where a greater proportion of patients in the astodrimer groups (compared with placebo) met the criteria (Table 2).

**Fig 2:**
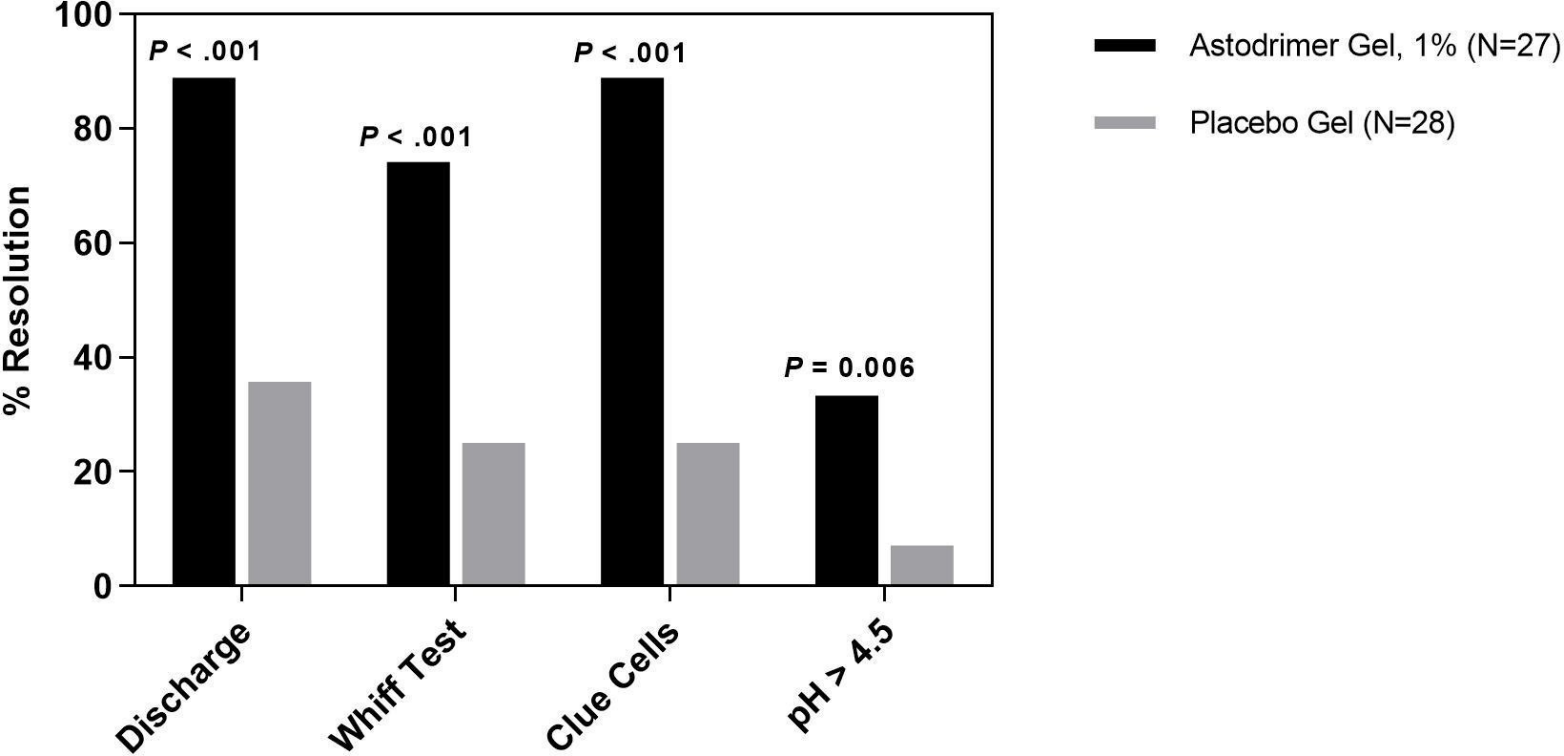
Resolution of Amsel criteria at day 9–12. Resolution of individual Amsel criteria (discharge, whiff test, clue cells and pH >4.5) at day 9–12, by treatment group (astodrimer 1%: N = 27; placebo: N = 28) (mITT population).

**Fig 3:**
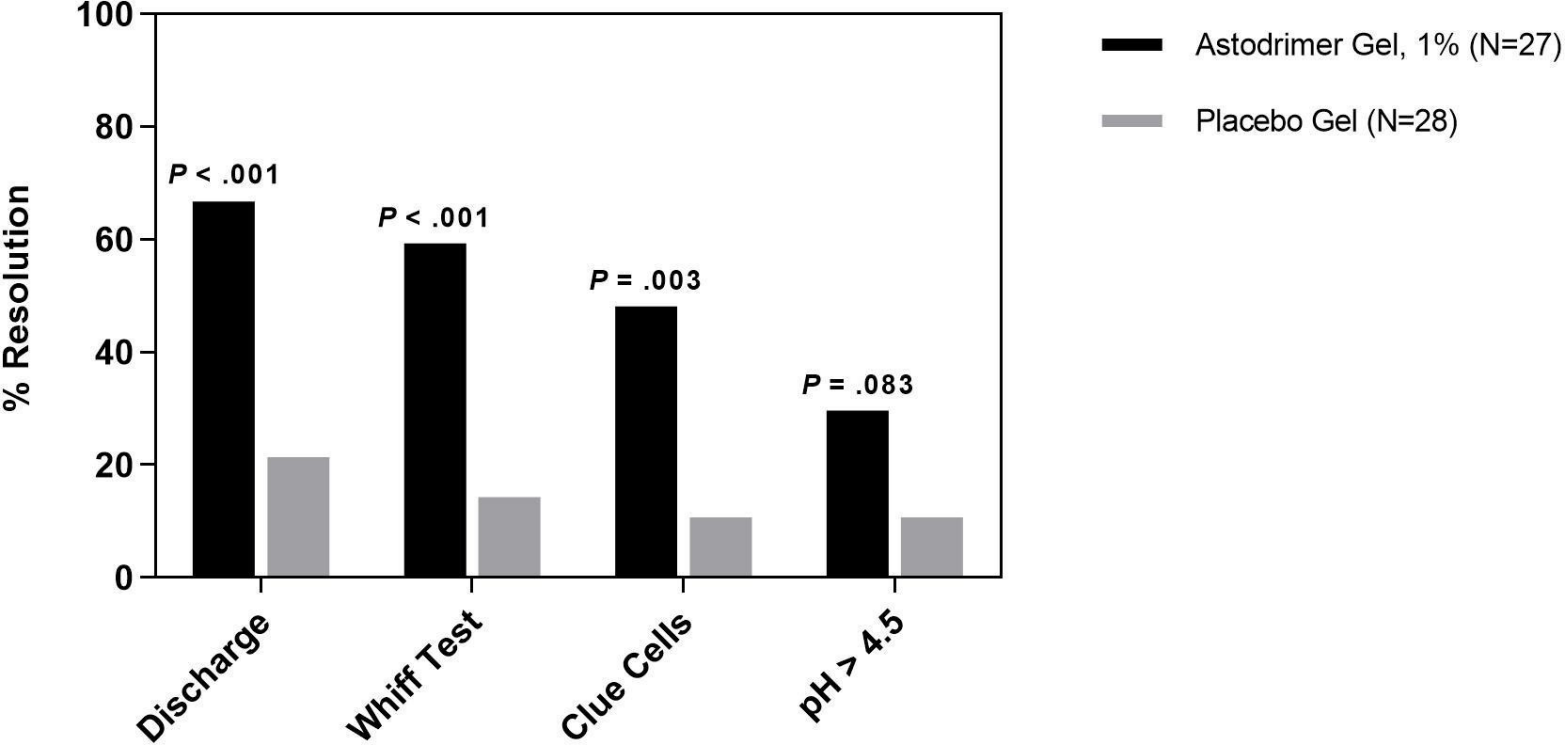
Resolution of Amsel criteria at day 21–30. Resolution of individual Amsel criteria (discharge, whiff test, clue cells and pH >4.5) at day 21–30, by treatment group (astodrimer 1%: N = 27; placebo: N = 28) (mITT population).

Significantly more patients treated with astodrimer experienced resolution of each individual Amsel criteria compared to those treated with placebo. Abnormal discharge resolved from baseline to day 9–12 for 88.9% of patients in the 1% astodrimer group compared to 35.7% of placebo patients (*P* < .001), positive whiff test resolved for 74.1% vs. 25.0% (*P* < .001), pH resolved for 33.3% vs. 7.1% (*P* = .006) and clue cells resolved for 88.9% vs. 25.0% (*P* < .001) (Fig 2). At day 21–30, a similar pattern was observed (Fig 3).

At baseline, most patients in all treatment groups had a NS categorized as ʽBVʼ. At day 9-12, the NS was categorized as ‘normal’ for 7.4% of placebo-treated patients compared to 28.0%, 26.9% and 27.6% of patients who were treated with astodrimer (3%, 1% and 0.5% doses, respectively, *P* < .001). Whilst most placebo-treated patients remained categorized as ‘BV’ at day 9–12 (88.9%), fewer than one-third of astodrimer-treated patients remained ‘BV’, regardless of dose (28.8%, 23/83; *P* < .001). Similar results were observed at day 21–30, although differences between the astodrimer and placebo groups were not statistically significant.

Both the investigator and patient symptom questionnaire responses supported the other efficacy findings. At day 9–12 and day 21–30, there were more patients who reported absence of symptoms of discharge and odor in the astodrimer groups (most notably the 1% dose) compared to placebo. At day 9–12, 63.0% and 77.8% of women given 1% astodrimer reported absence of vaginal discharge and absence of vaginal odor, respectively, compared to 25.0% and 28.6%, respectively, for placebo. Most women in the astodrimer groups also reported absence of discharge and odor at day 21–30. For the 1% dose group at day 21–30, 63.0% and 59.3% had absence of discharge and odor, respectively, compared with 25.0% and 25.0%, for placebo.

Time to first absence of discharge, odor, and discharge and odor was somewhat shorter in the astodrimer groups compared with placebo. When considering only responders (i.e., meeting requirements for clinical cure at day 21–30) the median [95% confidence interval {CI}] times to first resolution were: i) vaginal odor: 1 [0, 6] day for the 1% dose compared with 7 [0, 19] days for placebo; ii) vaginal discharge: 4 [2, 8] days for the 1% dose compared with 9 [0, 21] days for placebo; and iii) vaginal discharge and odor: 5.5 [2, 11] days for the 1% dose compared with 9 [0, 21] days for placebo.

Rescue medication (typically oral metronidazole or tinidazole) was needed by fewer women who received 3%, 1% and 0.5% astodrimer (50.0%, 25.9%, and 56.7%, respectively) than those who received placebo (75.0%). In the astodrimer groups, of those patients who received rescue medication, most did so at the day 21–30 visit. For placebo, uptake of rescue medication was similar at the day 9–12 visit, the day 21–30 visit, and between the two visits.

### Acceptability

The TSQM results supported other efficacy findings. At day 9–12, mean scores were greater for women receiving 3%, 1% and 0.5% astodrimer compared to placebo for effectiveness (68.6 [*P* < .001], 69.1 [*P* < .001] and 60.4 [*P =* .017], respectively, vs. 45.5 placebo) and Global Satisfaction (69.5 [*P* = .002], 75.0 [*P* < .001] and 61.2 [*P* = .042], respectively, vs. 46.6 placebo). The highest scores were observed for the 1% astodrimer group. Scores for convenience and tolerability were comparable between the astodrimer and placebo groups.

### Vaginal microflora culture analysis

The number of patients with *G*. *vaginalis*-positive cultures was reduced by more than half at day 9–12 compared to baseline in each of the astodrimer groups (e.g., from 87.9% [29/33] to 42.4% [14/33] in the 1% dose group). The reduction in the placebo group was modest: from 87.9% (29/33) to 72.7% (24/33). The number of women with *G*. *vaginalis* growth at day 21–30 remained lower than at baseline in all treatment groups. Anaerobic Gram-negative rods (pigmented and non-pigmented) were also reduced by treatment with astodrimer, with greater decreases compared to placebo for all astodrimer groups at day 9–12. The treatment appeared benign with respect to changes in *Lactobacillus* spp. There was no increase, and in fact a minor decrease, in the number of women culture positive for *Candida* spp. at day 9–12 and day 21–30 compared with baseline.

### Safety/tolerability

The overall incidence of AEs was 71.9% (23/32) for placebo vs. 65.6% (61/93) for the astodrimer groups (Table 3). During the treatment phase, AE incidences were 56.2% (18/32) and 51.6% (48/93) for women administered placebo and astodrimer, respectively. During follow-up, these incidences were 50.0% (16/32) and 41.9% (39/93), respectively. AE incidence in patients administered 1% astodrimer was the lowest among the groups.

**Table 3:** Tolerability by treatment group (safety population).

Parameter	3% Astodrimer N = 29 n (%)	1% Astodrimer N = 32 n (%)	0.5% Astodrimer N = 32 n (%)	Any Astodrimer N = 93 n (%)	Placebo N = 32 n (%)
Patients with ≥1 AE	20 (69.0)	19 (59.4)	22 (68.8)	61 (65.6)	23 (71.9)
Patients with ≥1 AE considered by investigator to be potentially related to study treatment	9 (31.0)	6 (18.8)	8 (25.0)	23 (24.7)	7 (21.9)
Patients with ≥1 severe AE	0	0	0	0	2 (6.3)
Patients with ≥1 serious AE	0	0	0	0	0
Patients with ≥1 GUAE considered by investigator to be potentially related to study treatment	7 (24.1)	6 (18.8)	8 (25.0)	21 (22.6)	7 (21.9)
Patients who stopped treatment due to AE	1 (3.4)	1 (3.1)	0	2 (2.2)	0
AE of special interest					
Vulvovaginal candidiasis/mycotic infection	3 (10.3)	2 (6.3)	2 (6.3)	7 (7.5)	1 (3.1)
Urinary Tract Infection/Cystitis	0	1 (3.1)	0	1 (1.1)	2 (6.3)
Metrorrhagia	0	0	2 (6.3)	2 (2.2)	0
Vulvovaginal erythema	0	1 (3.1)	0	1 (1.1)	0
Vulvovaginitis	1 (3.4)	2 (6.3)	1 (3.1)	4 (4.3)	1 (3.1)
AEs considered by investigator to be potentially related to study treatment (incidence ≥5%)					
Vulvovaginal pruritus	2 (6.9)	2 (6.3)	4 (12.5)	8 (8.6)	4 (12.5)
Vulvovaginal burning sensation	2 (6.9)	2 (6.3)	1 (3.1)	5 (5.4)	0
Vulvovaginal candidiasis/mycotic infection	3 (10.3)	1 (3.1)	1 (3.1)	5 (5.4)	1 (3.1)
Metrorrhagia	0	0	2 (6.3)	2 (2.2)	0

AE = adverse event, GUAE = genitourinary adverse event, N = number of patients analyzed, n = number of patients with observation

Incidence of AEs potentially related to study treatment was 24.7% (23/93) for the astodrimer groups vs. 21.9% (7/32) for placebo. Patients administered 1% astodrimer had an AE incidence of 18.8% (6/32), the lowest among the treatment groups. Genitourinary AEs (GUAEs) potentially related to treatment were reported in women administered astodrimer (22.6% [21/93]) at a similar rate to those given placebo (21.9%, [7/32]).

Just 1 patient (3.1%) in the 1% dose group and 1 patient (3.1%) in the placebo group experienced non-GUAEs potentially related to treatment (lethargy and headache, respectively). AEs of special interest (candidiasis [or vulvovaginal mycotic infection]), UTI [or cystitis], metrorrhagia, vulvovaginitis and vulvovaginal erythema) were infrequent. The rate of vulvovaginal candidiasis considered by the investigator to be potentially related to study treatment in the 1% astodrimer group was 3.1% (1/32) and 3.1% (1/32) for placebo.

Most AEs were mild or moderate in intensity. Severe AEs were only reported for 2 women, both of whom were placebo-treated. There were 2 AEs leading to treatment discontinuation; dysmenorrhea in the 3% astodrimer group, and sinusitis in the 1% astodrimer group; neither was considered related to study treatment. There were no serious AEs (SAEs).

## Discussion

Astodrimer is a new agent from the class of compounds called dendrimers. Astodrimer features a large molecular size and negative charge, which prevent systemic absorption and interfere with the ability of bacteria to adhere to surfaces inhibiting formation of and disrupting biofilms. A new therapy for the treatment of BV that avoids issues of conventional antibiotics, such as systemic exposure, candidiasis and antibiotic resistance, and has a novel mechanism of action targeting biofilms, offers a promising potential treatment modality for BV.

In this study, the clinical cure rate for 1% astodrimer at day 9–12 (74.1%) compares favorably to cure rates reported for conventional antibiotic products such as oral secnidazole (57.9%) [21] and metronidazole 1.3% gel (46%) [22] using comparable cure criteria assessed at a similar timeframe. The cure rate also compares favorably with BV cure rates obtained with an experimental boric acid/ethylenediamine tetra acetic acid (EDTA) vaginal gel intended to target biofilm in BV (50%) [23].

The median time to resolution of vaginal odor after starting treatment with Astodrimer Gel was 1 day. This result also compares favorably with a median time to resolution of vaginal odor for metronidazole gel, 1.3% and 0.75%, of 2 and 3 days, respectively, suggesting a more rapid relief of the highly problematic symptom of vaginal odor for Astodrimer Gel, 1%, compared with conventional antibiotics [24]. The findings of rapid and maintained symptom resolution are important in the context of the clinical management of BV, which is driven by presence and resolution of symptoms.

Differences between the astodrimer groups and placebo narrowed at the day 21–30 visit but clinical cure, the primary endpoint of the study, remained statistically significantly higher in the 1% astodrimer group compared with placebo. The clinical cure rate of 46.2% at day 21–30 is comparable to oral secnidazole using comparable cure criteria at a similar timeframe [21], and compares favorably with a study of metronidazole 0.75% gel showing that the clinical cure rate at day 21–30 was 28.8% [24].

Despite the narrowing of differences, there was a lower use of rescue medication by women receiving astodrimer in this study, and the non-antibiotic mechanism and well tolerated safety profile of Astodrimer Gel means that the product may be better-suited than standard antibiotic therapies for repeat courses, and for longer-term maintenance or preventive therapy in cases where that is required [25].

The safety profile of astodrimer was reflective of a topically applied, non-systemically absorbed product. No severe AEs were reported for astodrimer treated patients. Two AEs led to treatment discontinuation but neither case was considered treatment related.

Notably, the incidence of candidiasis for Astodrimer Gel, 1%, (6.3%, 2/32) was less than half that reported for secnidazole (13.6%, 17/125) [21].

Astodrimer was associated with positive changes in vaginal microflora as assessed by semiquantitative culture analysis. Culture and identification of bacteria from vaginal specimens is challenging, so the results of this aspect should be interpreted with caution [26]. Overall, however, the changes in astodrimer groups appeared to be associated with BV cure and improved symptoms, while potentially related AEs, such as candidiasis and UTI, were reported infrequently.

That the 1% dose achieved the best results in this study was somewhat surprising but is consistent with the hypothesis that the treatment of BV with astodrimer restores the vaginal flora to a more normal balance; therefore, a lower dose could fail to exert enough antimicrobial activity, while a higher dose could have an inhibitory effect in certain beneficial bacterial species, such as lactobacilli. This effect has been observed with another topically applied therapy, rifaximin, where a mid-range dose achieved highest rates of cure of BV [27].

### Strengths and limitations

Strengths of the study are that it was double-blind, multicenter, placebo controlled, and randomized. The study was adequately powered to detect a difference in BV cure rates between astodrimer and placebo.

A limitation of the study design was that randomization was not stratified for study center, since some assessments were investigator based, nor for women’s prior BV history, since number of prior episodes may represent a prognostic factor in the resolution of BV.

## Conclusions

Astodrimer Gel once daily for 7 days was superior to placebo for treatment of BV, was well-tolerated, and provided rapid resolution of BV symptoms. Patients found the treatment convenient and tolerable, and superior to placebo with respect to overall satisfaction and perceived efficacy. Clinical cure rates were generally favorable or comparable to those reported with conventional antibiotics.

These findings demonstrate the potential clinical utility of Astodrimer Gel, 1%, as an alternative treatment for BV with a novel mechanism of action related to disruption of biofilms. The product acts locally and is not systemically absorbed, offering patients and clinicians an additional safe treatment option that avoids potential issues associated with existing conventional antibiotics, such as high rates of candidiasis and antibiotic resistance. The 1% astodrimer dose consistently showed the strongest efficacy across primary and secondary endpoints, and was selected for assessment in phase 3 studies to confirm efficacy and safety findings [28].

## Supporting information

S1 Checklist CONSORT checklist. (DOC)

S1 Dataset Analysis populations. (XLSX)

S2 Dataset Efficacy outcomes. (XLSX)

S3 Dataset Symptom outcomes. (XLSX)

S4 Dataset Safety. (XLSX)

S1 Data Study protocol. (PDF)
