# Supplementary material for: A phase 2, double-blind, multicenter, randomized, placebo-controlled, dose‑ranging study of the efficacy and safety of Astodrimer Gel for the treatment of bacterial vaginosis
Source: PLoS One. 2020 May 4;15(5):e0232394. doi: 10.1371/journal.pone.0232394 (PMC7197797; doi:10.1371/journal.pone.0232394)
Supplement: S1 Data — (PDF) [file pone.0232394.s006.pdf]

A double-blind, multi-center, randomized, placebo controlled, dose-ranging study to determine the efficacy and safety of SPL7013 Gel (VivaGel®) administered vaginally in the treatment of bacterial vaginosis

**Protocol Number:**	SPL7013-013
**Investigational Product:**	SPL7013 Gel (VivaGel®)
**IND Number:**	XXXXXX
**Sponsors:**	Starpharma Pty Ltd<br>Baker Building<br>75 Commercial Road<br>Melbourne Victoria 3004<br>Australia
**Sponsors' Clinical Representatives:**	XXXXXXXX<br>Clinical Development Manager<br>Starpharma, Australia
**Principal Investigator:**	XXXXXXXX<br>Medical Monitor<br>Omnicare Clinical Research, USA
**Medical Expert:**	XXXXXXXX<br>Consultant in Genitourinary Medicine<br>Sheffield Hospital, UK
**Biostatistician:**	XXXXXXXX<br>Head of Technical Services, Asia Pacific<br>Omnicare Clinical Research, Australia

CONFIDENTIAL

This protocol includes information and data that contain trade secrets and privileged or confidential information which is the property of Starpharma Pty Ltd ("Starpharma"). This information must not be made public without written permission from Starpharma. These restrictions on disclosure will apply equally to all future information supplied to you. This material may be disclosed to and used by your staff and associates as may be necessary to conduct the clinical study.

**Version:** Final V3.0	**Date of Version:** 15th July 2010

TABLE OF CONTENTS

TABLE OF CONTENTS ii

PROTOCOL SIGNATURE PAGE vi

SITE PRINCIPAL INVESTIGATOR SIGNATURE PAGE vii

LIST OF ABBREVIATIONS viii

PROTOCOL SYNOPSIS x

1. PERSONNEL AND FACILITIES 2
1.1 Principal Investigator(s) 2
1.2 Study Sponsor 2
1.3 Biostatistics 2

2. INTRODUCTION 3
2.1 Background 3
2.2 Study Product 3
2.3 Primary Pharmacodynamics 4
2.4 Secondary Pharmacodynamics 4
2.5 Non-Clinical Safety 4
2.6 Clinical Studies 5
2.7 Rationale 6

3. STUDY OBJECTIVES 8
3.1 Primary Objective and Endpoint 8
3.2 Secondary Objectives and Endpoints 8

4. STUDY DESIGN 9

5. STUDY POPULATION 9
5.1 Target Population 9
5.1.1 Inclusion Criteria 9
5.1.2 Exclusion Criteria 10
5.1.3 Randomization Criteria 10
5.1.4 Number of Participants 11
5.1.5 Method of Assignment 11

6. STUDY PRODUCTS 11
6.1 Investigational Product 11
6.2 Dosage and Administration 11
6.2.1 Dose and Dose Justification 11
6.2.2 Administration 11
6.3 Supply, Packaging, Labeling and Storage 12
6.4 Treatment Allocation and Randomization 12
6.5 Emergency Code-Breaking Procedures 13

6.6 Dispensing and Accountability 13
6.7 Assessment of Adherence to Study Product 13
6.8 Rescue Medication 14
6.9 Other Concomitant Medication/Treatment 14
6.10 Precautions 15
6.11 Warnings 15

7. STUDY PROCEDURES AND EVALUATIONS 16
7.1 Assessment Periods and Study Procedures 17
7.1.1 Visit 1 - Screening Evaluation 17
7.1.2 Baseline/Enrolment (Visit 2, Day 1) 18
7.1.3 Visit 3 (Day 9-12) 19
7.1.4 Visit 4 (Day 21-30) – Test of Cure (TOC) 20
7.1.5 Interim Visits and Contact 20
7.1.6 Withdrawal Visit 20
7.1.7 Follow up Procedures for Female Participants Who Become Pregnant 21
7.2 Observation and Measurements: Assessment of Safety 21
7.2.1 Demographics and Medical, Surgical, Sexual, Menstrual History 21
7.2.1.1 Demographics 21
7.2.1.2 Medical and Surgical History 21
7.2.1.3 Sexual History 21
7.2.1.4 Menstrual History 22
7.2.2 Vital Signs / Physical Examination 22
7.2.3 Pelvic Examination 22
7.2.4 Adverse Events 23
7.2.5 Normal Vaginal Discharge 23
7.2.6 Laboratory Testing 24
7.2.6.1 STI Screen 24
7.2.6.2 Urinalysis 24
7.2.6.3 Assessment of BV 24
7.2.6.4 Assessment of Candidiasis 25
7.2.6.5 Pregnancy Test 25
7.2.6.6 Semi-Quantitative Analysis of Vaginal Flora 25
7.2.6.7 Sample for Storage 26
7.2.6.8 Handling and Processing of the Biological Specimens 26
7.2.6.9 Storage and Destruction of the Biological Specimens 26
7.2.7 Symptom Questionnaire 26
7.2.8 Acceptability Questionnaire 27
7.2.9 Diary Cards 27
7.3 Study Restrictions 27
7.3.1 Dietary 27
7.3.2 Confinement 27
7.3.3 Physical Activity 27
7.3.4 Sexual Activity 27
7.3.5 Concomitant Medication 27

8. SAFETY REPORTING 28
8.1 Adverse Event Definitions 29
8.1.1 Adverse Event (AE) 29

Starpharma Pty Ltd, Baker IDI Building, 75 Commercial Rd, Melbourne, Victoria 3004, Australia

8.1.2 Serious Adverse Event (SAE) 29
8.1.3 Unexpected Adverse Drug Reaction (UADR) 30
8.2 Reporting of an Adverse Event 30
8.2.1 Timeframe for Reporting Adverse Events 30
8.2.2 Timeframe and Timelines for Reporting Serious Adverse Events 31
8.3 Recording an Adverse Event 32
8.4 Clinical Laboratory Abnormalities and Other Abnormal Assessments as AEs or SAEs 34
8.5 Toxicity Management 35
8.6 Follow-Up of Adverse Events 35
8.7 Regulatory Reporting Requirements 35
8.8 IEC/IRB Reporting Requirements 36
8.9 Pregnancy 36
8.10 Safety Monitoring 36

9. CLINICAL MANAGEMENT 38
9.1 Participant Completion 38
9.2 Treatment Non-Responders 38
9.3 Study Participant Withdrawal 38
9.4 Procedures for Handling Withdrawals 39
9.5 Replacement of Withdrawn Participant 39
9.6 Lost to Follow-Up 39
9.7 Premature Termination of Study 39
9.8 Notification of Primary Care Physician 40

10. STATISTICAL CONSIDERATIONS 40
10.1 Review of Study Design 40
10.2 Criteria for Evaluation of Study Objectives 40
10.2.1 Primary Endpoints 40
10.2.2 Secondary Endpoints 40
10.2.3 Definition of Signs and Symptoms of Genital Irritation 41
10.2.4 Definition of Clinical, Nugent, Therapeutic Cure 41
10.2.4.1 Clinical Cure 41
10.2.4.2 Nugent Cure 41
10.2.4.3 Therapeutic Cure 41
10.2.4.4 Therapeutic Resolution of BV 41
10.2.5 Definition of “Treatment Non-Responders” 42
10.2.6 Description of Groups for Analysis 42
10.2.6.1 Description of Participant Completion 42
10.2.6.2 Randomized Dataset 42
10.2.6.3 Intent to Treat Safety Dataset 42
10.2.6.4 Safety Dataset 42
10.2.6.5 Per-Protocol (PP) Dataset 42
10.2.6.6 Microbiological Dataset 43
10.3 Exclusion of a Study Participant from Statistical Analysis 43
10.4 Sample Size Justification 43
10.5 Planned Interim Analysis 44
10.6 Randomization and Blinding Procedures 44
10.7 Safety Review 45
10.8 Statistical and Analysis Plan **Error! Bookmark not defined.**

10.9 Analysis of Efficacy 45
10.10 Analysis of Safety 46

11. HUMAN SUBJECTS PROTECTION 49
11.1 Regulatory Considerations 49
11.2 Independent Ethics Committee (IEC)/Institutional Review Board (IRB) 49
11.3 Interpretation of the Protocol / Protocol Amendments 49
11.4 Subject Informed Consent 50
11.5 Ethical Considerations 50
11.6 Confidentiality 50

12. ADMINISTRATIVE ASPECTS 51
12.1 Clinical Trial Agreement 51
12.2 Study File 51
12.3 Initiation of the Study 51
12.4 Participant Reimbursement 51
12.5 Participant Identification 51
12.6 Confidential Follow-up 52
12.7 Recording of Data 52
12.8 Monitoring of the Study 52
12.9 Protocol Deviations 53
12.10 Data Quality Control 54
12.11 Quality Assurance Audit/Inspection 54
12.12 Study and Site Closure 55
12.13 Record Retention 55
12.14 Study Report 56

13. INDEMNITY AND COMPENSATION 56
13.1 Compensation 56
13.2 Indemnity 56
13.3 Insurance 56

14. SPONSOR RESPONSIBILITIES 57
14.1 Funding 57
14.2 Supply of Study Materials and Study Documentation 57
14.3 Compliance with Regulatory Requirements 57
14.4 Transfer of Sponsor Obligations 57

15. USE OF DATA AND PUBLICATIONS 57

16. APPENDICES 58
16.1 Declaration of Helsinki 58
16.2 Toxicity Tables 59
16.3 Follow-up Evaluations 60
16.3.1 Clinical Evaluation upon Reporting GU Symptoms 60
16.3.2 Follow up of findings seen on pelvic exam 61
16.4 References 63

Starpharma Pty Ltd, Baker IDI Building, 75 Commercial Rd, Melbourne, Victoria 3004, Australia

PROTOCOL SIGNATURE PAGE

The signatures below constitute approval of this protocol and the attachments, and provide the necessary assurances that this trial will be conducted according to all stipulations of the protocol, including all statements regarding confidentiality, and local legal and regulatory requirements including ICH guidelines.

______________________________	______________________
XXXXXXXX<br>Principal Investigator<br>Omnicare Clinical Research	Date
______________________________	______________________
XXXXXXXX<br>Biostatistician, Head of Technical Services, Asia Pacific<br>Omnicare Clinical Research	Date
______________________________	______________________
XXXXXXXX<br>VP, Development and Regulatory Affairs<br>Starpharma Pty Ltd	Date
______________________________	______________________
XXXXXXXX<br>Clinical Development Manager<br>Starpharma Pty Ltd	Date
______________________________	______________________
XXXXXXXX<br>Consultant in Genitourinary Medicine<br>Department of GU Medicine<br>Royal Hallamshire Hospital and Sheffield Teaching Hospitals	Date

SITE PRINCIPAL INVESTIGATOR SIGNATURE PAGE

By signing this Protocol, the Investigator(s) acknowledges and agrees:

The Protocol contains all necessary details for conducting the study. The Investigator will conduct this study as detailed herein, in compliance with Good Clinical Practice[1] (GCP) and the applicable regulatory requirements, and will make every reasonable effort to complete the study within the time designated.

The Protocol and all relevant information on the drug relating to pre-clinical and prior clinical experience, will be made available (as the Investigators' Brochure[7]) to all physicians, nurses and other personnel who participate in the conduct of this study. The Investigator will discuss this material with them to assure that they are fully informed regarding the drug(s) and the conduct of the study.

This document contains information that is privileged or confidential. As such, it may not be disclosed unless specific prior permission is granted in writing by Starpharma Pty Ltd or such disclosure is required by federal or other laws or regulations. Persons to whom any of this information is to be disclosed must first be informed that the information is confidential. These restrictions on disclosure will apply equally to all future information supplied, which is indicated as privileged or confidential.

Starpharma Pty Ltd will have access to any source documents from which Case Report Form (CRF) information may have been generated. The Case Report Forms and other data pertinent to this study are the property of Starpharma Pty Ltd, who may utilize the data in various ways, such as for submission to government regulatory authorities, or in publication of the results of the study.

____________________________________ ____________________

Principal Investigator (Site) Signature Date

____________________________________ ____________________

Principal Investigator Name Site

Starpharma Pty Ltd, Baker IDI Building, 75 Commercial Rd, Melbourne, Victoria 3004, Australia

LIST OF ABBREVIATIONS

AE	Adverse Event / Adverse Experience
AIDS	Acquired Immunodeficiency Syndrome
Alpha (α)	Type 1 error
Amsel's Criteria	Criteria relevant to the diagnosis of BV as outlined by Amsel et al (1999)
API	Active Pharmaceutical Ingredient
BV	Bacterial Vaginosis
BUN	Blood Urea Nitrogen
CFR	Code of Federal Regulations
CIB (IB)	Clinical Investigators' Brochure (Investigators' Brochure)
Clinical Cure	Resolution of abnormal discharge and at least 2 out of the remaining 3 Amsel's criteria
CRF	Case Report Form
CRO	Contract Research Organization
DAIDS	Division of AIDS
FDA	Food and Drug Administration
GCP	Good Clinical Practice
GLP	Good Laboratory Practice
HEC Placebo Gel	Hydroxyethyl cellulose Placebo Gel
HIV	Human Immunodeficiency Virus
HSV-1 or -2	Herpes Simplex Virus (Type 1 or 2)
IB (CIB)	Investigators' Brochure (Clinical Investigators' Brochure)
ICF	Informed Consent Form
ICH	International Conference on Harmonization
IEC	Independent or Institutional Ethics Committee
IND	Investigational New Drug
IRB	Institutional Review Board
ITT	Intent to treat
KOH	Potassium Hydroxide
MedDRA	Medical Dictionary for Regulatory Activities
MHRA	Medicines and Healthcare Products Regulatory Agency
MOP	Manual of Procedures
N	Number (typically refers to participants)
NOEL	No Observed Effect Level
Nugent Cure	Cure defined as absence of BV by the Nugent Symptom Scoring method
Pap	Papanicolaou test

PIN	Participant Identification Number
PCR	Polymerase Chain Reaction
PBS	Phosphate Buffered Saline
PI	Principal Investigator
PK	Pharmacokinetics
PP	Per Protocol
QA	Quality Assurance
QC	Quality Control
RTI	Reproductive tract infection
SAE	Serious Adverse Event
SHIV	Chimeric Simian Human Immunodeficiency Virus
SOP	Standard Operating Procedure
SR	Selected Regimen
Starpharma	Starpharma Pty Ltd
STD	Sexually Transmitted Disease
STI	Sexually Transmitted Infection
Therapeutic Cure	Cure of BV defined as a composite of "Clinical Cure" and "Nugent Cure"
Therapeutic Resolution	"Clinical Cure" and a shift to intermediate or normal flora according to the Nugent scoring criteria
TOC	Test of Cure
UADR	Unexpected Adverse Drug Reaction
USA	United States of America
UTI	Urinary tract infection
WHO	World Health Organization

PROTOCOL SYNOPSIS

**Study Title:**	A double-blind, multi-center, randomized, placebo controlled, dose-ranging study to determine the efficacy and safety of SPL7013 Gel (VivaGel®) administered vaginally in the treatment of bacterial vaginosis
**Primary Objective:**	• To determine the clinical efficacy of 0.5%, 1% and 3% SPL7013 Gel compared to placebo gel in the treatment of BV
**Secondary Objectives:**	• To explore the microbiological and overall efficacy of 0.5%, 1% and 3% SPL7013 Gel compared to the HEC placebo gel<br>• To determine the safety and tolerability of SPL7013 Gel in the study population<br>• To determine Investigator-assessed and patient perceived symptom resolution and acceptability of SPL7013 Gel in the study population
**Hypothesis:**	SPL7013 Gel is effective for the treatment of BV
**Primary Endpoint:**	"Clinical Cure" at the Test of Cure (TOC) visit as defined by no abnormal discharge as described by the Amsel's criterion for vaginal discharge, and fulfilling no more than one of the other three criteria.
**Secondary Endpoints:**	• "Nugent Cure", "Therapeutic Resolution of BV" and "Therapeutic Cure" at the TOC visit<br>• "Clinical Cure", "Nugent Cure", "Therapeutic Resolution of BV" and "Therapeutic Cure" at Day 9-12 visit<br>• Investigator assessed and patient perceived symptom resolution<br>• Genital AEs possibly, probably, definitely related to study product<br>• All other AEs<br>• Treatment acceptability<br>• Association between BV signs and symptoms and clinical diagnosis of BV<br>• Semi-quantitative analysis of vaginal flora (sub-set only)
**Study Design:**	Multi-center, randomized, dose-ranging, placebo-controlled.
**Sample Size:**	132 women (N=33 per arm for 28 evaluable)
**Study Population:**	Women aged 18-45 years with a diagnosis of bacterial vaginosis by reported symptoms, Amsel's criteria, a Nugent score of ≥4, and otherwise healthy.
**Duration/Participant:**	Total duration of approx. 5-6 weeks comprising: up to a 1 week screening period; a 1 week treatment period; and a 3-4 week follow-up period.
**Study Procedures:**	After a maximum 1 week screening period, participants will be randomized to receive either 0.5%, 1% or 3% SPL7013 Gel or HEC placebo gel at a dose of 5g each night administered vaginally for 7 consecutive days. Participants will be assessed for BV (both clinically by Amsel's criteria and microbiologically by Nugent score) at screening, baseline (screening and baseline may be combined), after last

	application (Day 9-12) and at the final study visit (Test of Cure, TOC visit) approximately 2-3 weeks after last dose (Day 21-30).<br>At each visit participants will undergo: pelvic/gynecological examination, during which cervical and vaginal swabs will be taken (including a swab for a storage sample); adverse events will be recorded; and adherence to the protocol will be assessed. Acceptability Questionnaires will be administered at the end of the treatment period (Visit 3, Day 9-12), and Symptom Questionnaires at each visit. Participants will complete Diary Cards on a daily basis to capture data on study drug administration and compliance, and adverse events.<br>Women who report no improvement in their BV-associated discharge by Visit 3 (Day 9-12), and wish to receive further treatment will be offered rescue therapy in line with local practice, after all visit assessments have been completed. Rescue therapy may also be offered to women who relapse.
**Safety Parameters:**	Urinalysis, physical and pelvic/gynecological examination, medical history, AEs (as graded by the DAIDS toxicity tables), semi-quantitative analysis of vaginal flora (sub-set only).
**Statistical Analyses:**	Primary analysis will be a comparison of 0.5%, 1% or 3% SPL7013 Gel vs. placebo gel. Assuming Clinical Cure rates of 50% and 15% for SPL7013 Gel and placebo gel, respectively, a sample size of a minimum of 28 evaluable subjects per treatment arm will provide 80% power to detect a treatment difference with an alpha significance level of 0.05.<br>When 50% of patients have completed TOC at Day 21-30 visit, an interim analysis will be conducted to provide guidance in the decision of which dose groups based on preliminary efficacy and safety to include in a forthcoming protocol. The interim analysis will be carried out using the O'Brien-Fleming method with α=0.005 for the interim analysis and the significance level calculated for the final analysis being 0.048.

Table 1 Schedule of Study Assessments

Procedure	May be combined: Visit 1 (Day -7-0) **Screening**	May be combined: Visit 2 (Day 1) **Baseline**	Visit 3 (Day 9-12) **End of Treatment**[1]	Visit 4 (Day 21-30) **Test of Cure (TOC)**[2]
Informed Consent	x			
Demography	x			
Inclusion/exclusion criteria	x	x		
Medical/Surgical/Sexual / Menstrual History	x			
Vital Signs	x			
Physical Examination	x[3]	x[4]	x[4]	x[4]
Urine pregnancy test	x	x		x
STI assessment[5]	x	x[4]	x[4]	x[4]
Urine dip stick for UTI detection	x	x	x	
Pelvic exam with vaginal sampling[6]	x	x	x	x
Vaginal sample for semi-quantitative analysis [7]		x	x	x
Assessment of BV (Amsel's criteria)	x	x	x	x
Gram stain slide for Nugent's score	x	x	x	x
Visit to unit	x	x	x	x
Randomization		x		
Instruction in use of study product	x	x		
Dispense applicators		x		
Return used/unused applicators			x	
Issue diary card		x	x	
Return diary card			x	x
AE collection	x[8]	x[8]	x	x
Complete Symptom Questionnaire	x	x	x	x
Complete Acceptability Questionnaire (TSQM)			x	
Prior/Concomitant Meds	x[9]	x[9]	x	x

[1] Study participants who require rescue therapy must complete all assessments prior to administration of rescue medication.
[2] Procedures identical for Withdrawal Visit, but also includes completion of the Acceptability Questionnaire.
[3] Only to assess a stable but pre-existing condition at the investigator's discretion
[4] Only if required to assess/diagnose an emergent AE
[5] STI testing: Chlamydia and gonorrhoea according to standard local procedures; trichomoniasis by wet mount slide
[6] Including: vaginal sampling for dry and wet mount slides for Gram stain, trichomoniasis, clue cells and/or yeast; speculum exam of vagina and cervix; naked eye exam of vulva; assessment of pH; Pap smear (if required); vaginal swab for storage sample. A local test for BV may be performed to determine eligibility prior to receipt of Nugent symptom score.
[7] Vaginal samples for semi-quantitative culture analysis of vaginal flora – conducted in a sub-set only
[8] AEs that have been reported as starting prior to first application of study medication to be recorded in Medical History
[9] Concomitant medications that have been reported as starting prior to first application of study medication to be recorded under Prior Medication

1. PERSONNEL AND FACILITIES

Full contact details are provided in the Study Contacts List.

1.1 Principal Investigator(s)

XXXXXXXX
Medical Monitor
Omnicare Clinical Research
630 Allendale Road,
King of Prussia, PA 19406
USA
Tel: +1-484-679-3303
24hr Medical Monitor Phone Line: +1-800-836-2165
Fax: +1-800-252-2535

1.2 Study Sponsor

Starpharma Pty Ltd
Level 6, Baker IDI Building
75 Commercial Road
Melbourne, VIC 3004
Australia
Tel: +61 3 8532 2700
Fax: +61 3 9510 5955

1.3 Biostatistics

XXXXXXXX
Head of Technical Services, Asia Pacific
Omnicare Clinical Research
13-15 Lyon Park Road
North Ryde, NSW 2113
Australia
Tel: +61 2 9889 0500
Fax: +61 2 9889 0511

2. INTRODUCTION

2.1 Background

BV is the most common vaginal infection worldwide, and the most common cause of vaginal irritation, discharge and malodor. The condition is characterized by an imbalance of the normal vaginal microflora, with normally plentiful peroxide-producing bacteria (*Lactobacillus* spp.) being reduced in number or absent, and other anaerobic bacteria being abundant (*e.g., Gardnerella vaginalis*). The cause of BV remains unclear. However, given the epidemiology of BV is similar to that of well known STIs, it has been hypothesized that the condition may be the result of sexual transmission of an organism such as *G. vaginalis*[2], although this is still uncertain.

Both symptomatic and asymptomatic BV have been linked to serious public health consequences, including pre-term birth[3], postoperative infection, and acquisition and transmission of HIV and other STIs.[4] Common therapies for BV are generally recognized by clinicians as being inadequate, and rates of relapse of symptoms of BV following therapy are high.[5] Therefore, new technologies and methodologies are urgently needed to both treat and prevent relapse of this potentially embarrassing condition.

SPL7013 has been shown to be active against *G. vaginalis*, *Bacteroides ovatus* and *Prevotella bivia*, which are all implicated in, or at least representative of genera implicated in, the pathogenesis of BV. In contrast to some existing treatments for BV, SPL7013 appears to be non-toxic to a wide range of *Lactobacillus* spp., with only minimal inhibition of growth being observed for one strain in vitro.

These microbiology data warrant clinical investigation of SPL7013 Gel as an alternative to current treatments for BV.

2.2 Study Product

SPL7013 is the active pharmaceutical ingredient (API) which is formulated into a vaginal gel, SPL7013 Gel, or VivaGel®.

SPL7013 is a member of the class of compounds called dendrimers, a chemically diverse array of macromolecules. Dendrimers are characterized by multiple layers of subunits branching out from a central core synthesized by repeated stepwise addition of branching units to a core.[6] As pharmaceuticals, dendrimers offer a unique single-molecule structure for the presentation of multiple copies of a given surface group attached to the underlying dendrimer architecture.

Full details of the product (in terms of structure, formulation and mechanism of action) may be found in the Investigators' Brochure (IB).[7]

2.3 Primary Pharmacodynamics

The *in vitro* antimicrobial activity of SPL7013 has been investigated against a range of pathogens (including *G. vaginalis, B. ovatus* and *P. bivia*), which are all implicated in or at least representative of genera implicated in the pathogenesis of BV. Both unformulated SPL7013 and formulated SPL7013 Gel preparations were assayed in a range of studies, with comparator drugs including clindamycin, metronidazole, imipenem, amphotericin B and ciprofloxacin. Activity of SPL7013 against anaerobic bacteria and also yeast was investigated.

Unformulated SPL7013 was tested at concentrations relevant to that contained in the vaginal gel formulation, *i.e.* 1, 5, 10, or 30 mg/mL, equivalent to 0.1, 0.5, 1 and 3% (w/w) SPL7013 Gel, respectively. Some anaerobic pathogens were sensitive to either 30 mg/mL (*B. ovatus, P. bivia*), 10 mg/mL (*L. crispatus*), or 5 mg/mL (*G. vaginalis*) of SPL7013. There was no activity against yeast or *Neisseria gonorrhoeae* at these higher concentrations.

The observed inhibition of anaerobic bacteria at concentrations relevant to the 0.5, 1 and 3% (w/w) SPL7013 vaginal gel formulation suggests a species-specific, selective antimicrobial mechanism of action.

Full details of the studies and mechanisms of activity may be found in the IB.[7]

2.4 Secondary Pharmacodynamics

In vitro and *in vivo* studies in mice and guinea pigs on a selection of dendrimer-based compounds have reported potent inhibition of HSV-1 and HSV-2.[8,9,10] In addition, *in vitro* and *in vivo* studies of SPL7013 have demonstrated antiviral activity against HIV type 1 (EC_{50}=1.90 µg/ml), HIV type 2 (EC_{50}=4.38 µg/ml) and chimeric-simian human immunodeficiency viruses ($SHIV_{162P3}$ and $SHIV_{89.6P}$) (EC_{50}=0.25 µg/ml).[11] Contraceptive activity has also been demonstrated for SPL7013 in the rabbit model and *in vitro* testing, although it has been found to be not spermicidal. Full details of the studies and mechanisms of activity may be found in the IB.[7]

2.5 Non-Clinical Safety

XXXXXXXXXXXXXXXXXXXXXXXXX of vaginal administration of SPL7013 Gel have suggested a low risk for acute toxicity, and a lack of evidence for systemic toxicity.

Repeated vaginal administration of SPL7013 Gels (0% to 5%) to multiple species generally produced a low grade response. There was no clear indication from any of the studies of a potential safety concern for humans.

Studies of XXXX administration XXXXX and rectal administration XXXXXXXXXX indicate that 3% w/w SPL7013 Gel is well tolerated on both the penile epithelium and rectal mucosa.

Starpharma Pty Ltd, Baker IDI Building, 75 Commercial Rd, Melbourne, Victoria 3004, Australia

Oral single and repeat dose toxicology studies in XXXX suggest a low risk of acute toxicity from SPL7013, with very limited systemic absorption. Studies investigating intravenous administration of SPL7013 in XXXXXXXX demonstrated signs of toxicity (both clinically and on necropsy) in some animals at the higher dose levels. The NOEL was determined to be 25 mg/kg for the intravenous administration route.

In vitro studies of cytotoxicity suggest that SPL7013 has a significant therapeutic index, with little potential for cytotoxicity. Negative findings in an Ames test, *in vitro* mammalian chromosome aberration assays (Chinese hamster ovary cells) and a rat bone marrow erythrocyte micronucleus test, indicate that SPL7013 has no genotoxic potential.

The effect of SPL7013 Gel on latex condoms has been assessed in a number of studies. The studies demonstrated that SPL7013 Gel did not compromise the integrity or affect the dimensions of non-lubricated, silicone lubricated, and aqueous lubricated condoms.

Further details of all studies are available in the IB.[7]

2.6 Clinical Studies

Clinical experience to date for SPL7013 is based on five completed clinical studies [SPL7013-001, SPL7013-002, SPL7013-003, SPL7013-004 and SPL7013-006 (MTN-004)]. All studies were double-blind, randomized and placebo-controlled (against vehicle-only gel), with the exception of Study SPL7013-003, which was open-label.

In SPL7013-001, seven days consecutive vaginal dosing with 3.5g SPL7013 Gel strengths of 0.5%, 1% and 3% (total daily dose range 17.5mg to 105mg) were investigated against placebo in a dose-ranging manner in healthy women volunteers. Healthy male volunteers were investigated in study SPL7013-002, where 2g of 3% w/w SPL7013 Gel (total daily dose of 60mg) or placebo was administered to the penis of healthy circumcised and uncircumcised men for seven consecutive days. During SPL7013-003 women were administered five single doses of 3% SPL7013 Gel with a wash-out of at least 5 days between doses. After each single dose a vaginal sample was taken at either Baseline (within 2-10 minutes), 1, 3, 12 and 24 hours. These samples were assessed for both anti-HIV and anti-HSV-2 activity using cell-based assays, and the mass and concentration of SPL7013 in samples was also determined. Administration of 3% w/w SPL7013 Gel or placebo gel twice daily (total daily dose of 210mg for active group) for 14 days was investigated in healthy, sexually abstinent women in study SPL7013-004 and in sexually active women in study SPL7013-006 (MTN-004). Safety evaluations conducted during these studies included: collection of signs and symptoms of genital irritation, collection of other adverse experiences (AE), and assessment of systemic absorption. Additional assessments in the female studies include colposcopy, assessment of vaginal microflora, and assessment of exploratory markers of inflammation.

No safety or tolerability issues have been flagged to date, nor serious AEs (SAEs) reported. Additionally, there have been no grade 3 or 4 AEs reported of either genital AEs or non-genital AEs, except for one report of severe tension headache in the placebo group in Study SPL7013-001. The majority of reports of genital irritation have been mild and similar in nature and incidence to those reported in comparable studies for other topical microbicides in development. The pharmacokinetic aspect of all completed studies showed that SPL7013 was not detected in any plasma samples analyzed. This finding suggests that SPL7013 is not absorbed into the blood following vaginal or penile administration of SPL7013 Gel.

Quantitative vaginal flora microbiology analysis in study SPL7013-001 showed a decreased concentration of *Lactobacillus spp.* and anaerobic gram negative rods (AGNR) with SPL7013 Gel. There was also an increase in facultative Gram-negative rods colonization rate seen in both active and placebo groups. Similar quantitative analysis of vaginal flora in study SPL7013-006 showed a decreased prevalence of *Lactobacillus* spp. and anaerobic gram negative rods (AGNR) with SPL7013 Gel. Analysis of flora microbiology in study SPL7013-004 however indicated that, although levels of *Lactobacillus* spp. were generally low in the population (as expected in a population mainly recruited from Kisumu, Kenya), persistence of both H_2O_2 producing and non-H_2O_2 producing *Lactobacillus* spp. was common throughout the course of the study, and did not differ from baseline nor between study arms. In study SPL7013-006, a decrease in prevalence and greater than one log decrease in concentration of *G. vaginalis,* and an approximate one log increase in the concentration of *Enterococcus* spp., Group B *Streptococcus* and coliforms was also observed with SPL7013 Gel. There was no overall impact on incidence of BV due to these apparent changes according to Nugent score.

The results from these studies suggest that there would be a low probability of safety and tolerability issues with the investigational product. The genital AEs reported in studies to date were mainly mild, with only occasional moderate genital AEs reported, and in these studies daily doses of 3% SPL7013 were administered up to twice daily for 14 days.

A full assessment of each completed clinical study is provided in the IB.[7]

2.7 Rationale

As noted above, SPL7013 has been shown to be active (*i.e.* inhibited growth) against *G. vaginalis*, *B. ovatus*, and *P. bivia*, which are all implicated in the pathogenesis of BV, while lacking effects against all but one strain of *Lactobacillus* tested. In other studies, SPL7013 has been shown to be non-toxic to *L. crispatus* and *L. jensenii*. These data suggest that SPL7013 Gel may have utility as either a treatment or preventative measure against BV, although there is a lack of an animal model in which to further explore this potential effect. Therefore, the intention of this study is to investigate whether SPL7013 Gel is effective as a treatment for BV, and if so, which gel strength(s) is suitable for further development.

The three gel strengths for investigation during this study have been selected based on microbiology data. The lower strength gel, 0.5%, has been selected as data indicates efficacy against *G. vaginalis* without affecting *L. crispatus*, the only *Lactobacillus* species in which SPL7013 has been shown to have activity. The higher gel strength, 3% has been selected as it provides the broadest activity (against *B. ovatus*, *P. bivia* and *G. vaginalis*), and the 1% strength selected to investigate a mid-point between the two.

Women with symptomatic BV have been specifically excluded from the clinical trials conducted to date; however, women with asymptomatic BV have not. The number of women with asymptomatic BV enrolled in to the completed studies of SPL7013 Gel has not been high; however, when Nugent scores have been seen to normalize in these women, it appears to occur after seven days of treatment. In addition, standard antibiotic therapy for BV is usually for 5-10 days, and seven days' administration of SPL7013 Gel has been shown to be safe and well tolerated up to a daily dose of 210mg SPL7013. One week has therefore been selected as the duration of treatment, with a maximum daily dose of 150mg SPL7013.

The primary endpoint of the study, resolution of BV according to the Amsel's criteria, which is mainly symptom based, reflects that women with BV are particularly distressed by the main clinical symptoms of the condition (*i.e.* odor and discharge). It is resolution of these symptoms that they seek primarily to resolve.

The HEC placebo (or "Universal Placebo") was selected as the comparator for this study as the gel has been specifically formulated and developed as a placebo for studies of vaginal products and is designed to have minimal irritancy potential, minimal effect on vaginal pH, and no efficacy in the treatment or prevention of disease. The HEC placebo has been validated as a vaginal placebo in two large Phase 3 studies in which over 3,000 women were exposed to the HEC placebo gel (HPTN-035[12] and MDP-301[13,14]). During both studies, the gel was safe, well tolerated, and lacked any efficacy against STIs, pregnancy and BV. Use of the HEC placebo gel will allow maintenance of the study blind and therefore will provide an objective study outcome.

If SPL7013 Gel is found to be effective in the treatment of BV, it would offer an alternative to current treatments, which are very limited in terms of both number as well as effectiveness. As SPL7013 Gel is locally acting, not systemically absorbed and, in contrast to products such as clindamycin vaginal cream, does not affect the integrity of non-lubricated, silicone lubricated, or aqueous lubricated condoms, it is an attractive candidate for further investigation.

3. STUDY OBJECTIVES

3.1 Primary Objective and Endpoint

The primary objective of the study is:

- To explore the clinical efficacy of 0.5%, 1% and 3% SPL7013 Gel in the treatment of BV compared to the HEC placebo gel.

This will be determined by comparison of the following Primary Endpoint:

- “Clinical Cure” at the Test of Cure (TOC) visit, defined as no abnormal discharge as described by the Amsel’s criterion and fulfilling no more than one of the other three remaining criteria.

3.2 Secondary Objectives and Endpoints

The secondary objectives of the study are as follows:

- To explore the microbiological and overall efficacy of 3% SPL7013 Gel in the treatment of BV compared to the HEC placebo gel.
- To determine the safety and tolerability of SPL7013 Gel in the study population.
- To determine the Investigator-assessed and patient perceived symptom resolution and treatment acceptability of SPL7013 Gel in the study population.
- To assess the association of BV signs and symptoms with clinical diagnosis of BV.

These will be determined by comparison of the following Secondary Endpoints:

- “Nugent Cure” (defined as a Nugent score of ≤3 when a score of 7 or more was determined at Baseline) at Visit 3 and the TOC visit.
- “Therapeutic Cure” (defined as a composite of both “Clinical Cure” and a Nugent score of 3 or less irrespective of status at Baseline) at Visit 3 and the TOC visit.
- “Therapeutic Resolution of BV” (defined as a Nugent score of ≤6 and fulfilling the criteria of “Clinical Cure”) at Visit 3 and the TOC visit.
- “Clinical Cure” at Visit 3.
- Genital AEs considered to be possibly, probably or definitely related to study product.
- All other AEs.
- Change from baseline in “Symptom Questionnaire” score at Visit 3 and TOC visit.
- “Acceptability Questionnaire” (Treatment Satisfaction Questionnaire for Medication (TSQM)) domain scores at Visit 3
- Correlation of Symptom Questionnaire responses with Nugent score at Visit 3 and TOC.
- Semi-quantitative analysis of vaginal flora (sub-set only)

4. STUDY DESIGN

This study is a double-blind, multi-center, randomized, placebo-controlled, dose-ranging study in women with a diagnosis of BV by Amsel's Criteria (all 4 criteria present) and a Nugent Score ≥4. After a maximum 1 week screening period, participants will be randomized to receive either 0.5%, 1%, 3% SPL7013 Gel or HEC placebo gel at a dose of 5g each night administered vaginally for 7 consecutive days. Participants will be assessed for BV (both by Amsel's criteria and by Nugent score) at screening, baseline, after last application on Day 7 (Day 9-12) and at the final study visit, the Test of Cure (TOC) visit approximately 2-3 weeks after last dose (Day 21-30).

At each visit participants will undergo: pelvic/gynecological examination, during which vaginal swabs will be taken (including a sample for storage); adverse events (AEs) will be recorded; a Symptom Questionnaire will be completed, and adherence to the protocol will be assessed. Acceptability questionnaires (for treatment satisfaction) will be administered after completion of treatment (Visit 3). Participants will complete Diary Cards on a daily basis to capture data on study drug administration and AEs.

The study is expected to be a maximum of 5-6 weeks in duration for each participant.

5. STUDY POPULATION

To assess any potential impact on participant eligibility with regard to safety, the investigator must refer to the IB[7] for detailed information regarding warnings, precautions, contraindications, AEs, and other significant data pertaining to SPL7013 Gels and placebo.

5.1 Target Population

The target population for recruitment is 132 women aged 18-45 years with a diagnosis of BV by Amsel's Criteria[15], who are otherwise healthy.

5.1.1 Inclusion Criteria

1. Women aged 18-45 years.
2. A diagnosis of BV defined as presentation with all of the four Amsel's Criteria[15] (*i.e.* all four of the following symptoms: presence of white to grey homogeneous discharge; positive whiff test (amine odor) with addition of potassium hydroxide (KOH); vaginal pH greater than 4.5; and presence of clue cells), a Nugent score[16] of ≥4, and patient reported symptoms.
3. Willing and able to provide written informed consent.
4. Healthy and with a regular menstrual cycle as assessed by medical history and medical interview.
5. If heterosexual or bisexual and of child-bearing potential, using an effective method of contraception and intention to use this method for the duration of study participation, including one month after cessation of study product. An "effective method of

contraception" is defined as: surgical sterilization; documented vasectomy of partner(s); intra-uterine device inserted at least 30 days prior to entry in to the study; or hormonal contraception (non-vaginally administered).

6. Able to understand and willing to comply with study protocol procedures and restrictions.
7. Reported negative Papanicolaou (Pap) smear in previous 2 years (or in line with local guidelines) for participants over 21 years old (if not available, screening will be offered in accordance with local clinical guidance).

5.1.2 Exclusion Criteria

1. Presence or history of allergy to topical vaginal products, or to SPL7013 Gel, HEC placebo gel or their components.
2. Abnormal pelvic examination at Baseline that, in the Investigator's opinion, indicates the participant is unsuitable for the study, including presence of vulvovaginitis and/or cervicitis.
3. Tests positive for Chlamydia, gonorrhea or trichomoniasis infection at screening.
4. Use of any vaginally administered products (including douches, personal lubricants and therapeutic products) other than the study products during the screening and study periods, other than the participant's usual brand of tampons.
5. Participation in any other drug or device study within 30 days or 5 half-lives which-ever is the longer, or has ever received VivaGel® in a previous study.
6. Any social or medical condition precluding enrolment in to the study.
7. Recent vaginal/cervical surgery (within 3 months of screening).
8. Pregnant, planning to become pregnant, or breast-feeding, or within 3 months of last pregnancy outcome at enrolment.
9. Use of oral and/or vaginal antibiotics or antifungals within 14 days of enrolment.
10. Tests positive for urinary tract infection (by urine dipstick) (women may be treated for their UTI and re-screened once the infection has fully resolved and exclusion criterion 9 is fulfilled).
11. The Investigator considers the participant to be a an increased risk of having an accompanying STI.
12. In the opinion of the investigator, should not participate in the study.

5.1.3 Randomization Criteria

All inclusion and exclusion criteria must be met by each participant before they can be randomized in to the study. An exception may be made if a local test for BV is performed during screening and provides a positive result, in such cases the participant may be enrolled pending result of the Nugent symptom score. If the score is later determined to be less than 4, the participant must be withdrawn.

5.1.4 Number of Participants

A total of 132 eligible women will be recruited in to the study, such that 112 are expected to be evaluable.

Drop-outs or withdrawals (as defined in Section 9) may be replaced at the discretion of the sponsor.

5.1.5 Method of Assignment

This study is a double-blind, randomized, placebo-controlled, dose-ranging study. Participants will be allocated a unique identification number (Participant Identification Number, PIN) at randomization from a list of numbers that will be assigned sequentially. The PIN will identify the treatment group to which she has been randomized, and the package of clinical trials supplies allocated to her. The randomization list will be held by an independent third party and will not be accessible to any of the study team until the blind has been broken.

6. STUDY PRODUCTS

6.1 Investigational Product

The investigational product is 5g SPL7013 Gel, containing 0.5%, 1% or 3%w/w SPL7013, packaged in single dose applicators. The HEC placebo gel will be packaged in identical applicators. The investigational formulation and all details on the study product are described in the IB.[7]

6.2 Dosage and Administration

6.2.1 Dose and Dose Justification

The three gel strengths 0.5%, 1% and 3% w/w SPL7013 have been selected for investigation in this study based on microbiology data. The lower strength gel, 0.5%, has been selected as data indicates efficacy against *G. vaginalis* without affecting *L. crispatus*, the only *Lactobacillus* species against which SPL7013 has been shown to have activity. The higher gel strength, 3% has been selected as it provides the broadest activity (against *B. ovatus*, *P. bivia* and *G. vaginalis*), and the 1% strength selected to investigate a mid-point between the two.

The duration of treatment in SPL7013-013 is once daily vaginal administration for 7 days and is based on previous experience showing that where Nugent scores normalize in clinical studies of SPL7013 Gel completed to date, this effect appears to occur after seven days' treatment. In addition, standard antibiotic therapy for BV is usually for 5-10 days

6.2.2 Administration

Study participants are to administer study gel product once daily, at bedtime.

Participants will be instructed to administer a single dose of study product by inserting the applicator in to the vagina and fully depressing the plunger slowly and evenly to ensure application of all gel. Used applicators are to be stored in a sealed plastic bag, and returned to the site at the next visit for compliance checking, after which they will be destroyed by the site.

Participants are asked to mark the time of application in their diary cards, and/or indicate any missed doses with reasons (if applicable). Instructions (including diagrams) in the correct application of the product will be provided to subjects during the Baseline visit and also included on the diary cards.

Unused applicators are to be returned to the study site.

6.3 Supply, Packaging, Labeling and Storage

The three active gels and the placebo gel are all colorless, clear gels and will be packaged in identical packaging so as to retain the study blind. Each applicator will be overwrapped in a sealed opaque envelope and labeled with the unique PIN. The product will be provided to participants in a single carton, which will contain eight identical overwrapped applicators and which will be labeled with the unique PIN. This carton will be dispensed to each participant at the Baseline Visit.

Product labeling will comply with FDA and local country requirements for Investigational Products.

The SPL7013 Gels and HEC placebo gel can be stored for up to 24 months, between 15-30°C (59-86°F), with short-term excursions permitted between 2-40°C (36-104°F) in storage/shipping. SPL7013 Gels have been shown to be stable in the vaginal applicators for up to 24 months at room temperature (25-30°C, 77-86°F) and up to 9 months at 40°C (104°F).

Study product is to be stored in a locked cupboard at the site, or within the pharmacy or another suitable limited-access area.

6.4 Treatment Allocation and Randomization

This study is a double-blind, randomized, placebo-controlled, dose-escalating study. Participants will be randomly allocated to receive one of: 0.5%, 1% or 3% SPL7013 Gel or HEC placebo gels, at a dose of 5g once daily at night for 7 days. Randomization will occur using a computer generated randomization list based upon a permutation block procedure.

The randomization list will link the treatment group to the unique PIN, which is allocated upon participant randomization. The computer generated randomization list employed will also be held by a third party, who is independent of the study team and study sites.

Each carton of study product will bear the PIN; this number will also be on the label of each applicator within the carton. The pharmacist/or designee will dispense the study product to the particular participant who has been allocated that PIN and the dispensing activity will be noted in the pharmacy records. A tear-off portion of the carton label is to be removed from the carton label and placed into the participant CRF. The tear-off portion will also contain a scratch-off area to be removed only in the case that unblinding is deemed necessary.

The randomization list will not be provided to the central laboratory determining the Nugent score.

6.5 Emergency Code-Breaking Procedures

There are no circumstances under which it is expected that unblinding will be necessary for the provision of medical treatment or to otherwise protect the safety of study participants. Study product can be withdrawn by the Investigator as described in Section 9.3. If an Investigator indicates that specific product knowledge is necessary to protect participant safety, the Investigator will notify the Safety Monitor to consider and rule upon the request. In the event that the request is granted, the Investigator may scratch-off the part of the label to reveal the treatment allocation. The full process of unblinding should be documented in the study file. If at all possible, other members of the study team should remain blinded to the treatment the participant received.

If code break becomes necessary during the study, Starpharma Pty Ltd and the Independent Ethics Committee (IEC) will be notified, and the date, time and reason will be recorded in the participant's records and CRF.

6.6 Dispensing and Accountability

Study product is to be requested *via* a product request form or prescription, which is to be signed and dated by the Investigator. The Investigator is responsible for ensuring accurate records of receipt of study products are retained, including dates of receipt. In addition, accurate records must be kept regarding when and how much of each study product was administered to each individual participant in the study.

At completion of the study all unused study product must be reconciled via detailed records itemizing all movement of study product to, from and within study site, and dispensing records.

6.7 Assessment of Adherence to Study Product

Adherence to the study product use regimen will be determined at Visit 3 via participant diary cards, which will capture frequency of gel use and stated reasons for non-use, and reconciled applicators. If there are discrepancies between reported adherence in the diary card and returned applicators, then the participant will be questioned further by site staff to determine which is accurate and the diary card/participant file updated accordingly.

Both used and unused applicators will be retained and returned to the study site at Visit 3 for compliance checking. The number of returned applicators will be recorded in the CRF, and an estimate of adherence recorded. Additionally, if sites have the capability, applicator staining will be employed to confirm exposure of the applicator to vaginal mucous, and confirm administration of the gel. A participant will be considered to be study drug compliant if she has administered at least 6 out of 7 doses of study product over a 7 day period.
Each participant will be counseled on the importance of using the study gel according to the study regimen at the Baseline Visit.

6.8 Rescue Medication

As it may take a couple of weeks for BV to fully resolve and the vaginal environment to return to normal, every effort should be made to retain study participants in the trial up to the final visit (Visit 4, TOC). However if participants are not satisfied that symptoms are improving, rescue medication/therapy may be provided if:

- at Visit 3, the study participant reports no improvement in BV-associated discharge and wishes to receive alternative therapy;
- after Visit 3 but prior to Visit 4, the study participant reports that symptoms have returned and wishes to receive alternative therapy.
- at Visit 4 the investigator determines that the BV has recurred, or study participant reports that symptoms have returned.

Prior to administration of rescue therapy, participants are to be assessed as per their next scheduled study visit (*i.e.* Visit 3 or Visit 4 (TOC)). Rescue medication will be the standard therapy for BV according to local practice at the study site. Details of any rescue medication provided to a study participant must be entered in to the CRF, and marked as such. Study participants who receive rescue therapy prior to Visit 4 (TOC) are considered to be "Treatment Non-Responders". Women who receive rescue therapy will continue to be assessed during the remaining study visits.

Medication may also be provided by the study participant's primary care physician after the participant has completed the study if her BV recurs after Visit 4. No details of any therapy received after completion of the study is to be provided.

6.9 Other Concomitant Medication/Treatment

Apart from rescue medication as outlined above, there are no concomitant medications that are to be specifically used as part of this study.

Vaginally administered products (lubricants, menstrual hygiene products (except tampons), contraception products, etc.) and oral antifungals are discouraged but should not lead to withdrawal if commenced during the study; however, the use of these is the basis for exclusion

criteria at Screening/Baseline. Antibiotics by any route are excluded during the on-study period unless provided for rescue therapy as per Section 6.8 as they will confound the study endpoints. Vaginally administered anti-fungals are also excluded during the on-study period. Women who receive antibiotic treatment (via any route) or vaginal antifungals during the study will cease study medication, but continue to be assessed during the remaining study visits. These women will not be included in the determination of the efficacy endpoints. Refer to Section 7.3.5 for more detail. Apart from concomitant medications specifically excluded under the inclusion/exclusion criteria, there are no other restrictions on concomitant treatments.

All concomitant medications administered from Screening to Follow-up must be recorded in the CRF.

6.10 Precautions

The Principal Investigator, Co- and Sub-Investigators and all study staff must familiarize themselves with the pre-clinical and clinical data to date as outlined in the IB.

6.11 Warnings

Based on the animal pharmacokinetic and toxicology data and the clinical data from the human studies, the following events may be experienced by women following application of SPL7013 Gel or HEC placebo gel:

- Metrorrhagia;
- Genitourinary erythema;
- Vaginal discharge of a curdled white material;
- Genitourinary discomfort/pain;
- Genitourinary pruritus;
- Dyspareunia;
- Burning sensation;
- Dysuria;
- Bacterial vaginosis;
- Headache;
- Respiratory Tract Infection;
- Lymphadenopathy.

This list represents the most commonly reported AEs observed during the clinical studies to date, irrespective of perceived causality to study product. Although not expected, other risks that are currently unknown may be associated with the treatment.

Systemic absorption of SPL7013 following vaginal administration of SPL7013 containing gels has not been observed in either animal or human studies.

The reproductive effects of SPL7013 Gel have not been investigated in clinical trials. The effects of SPL7013 Gel on the developing human fetus and breast-feeding child are also unknown. For these reasons, participants of childbearing potential must agree to use adequate contraception (*i.e.* hormonal contraceptive (but not vaginal rings), IUD, sterilization or vasectomy) prior to study entry and for the duration of the study. The chosen method of contraception must be fully effective upon entry in to the study. Women recruited in to this study will undertake a pregnancy test prior to the first dose of study medication and at the Baseline and final Follow-Up visits. Should a woman become pregnant or suspect that she is pregnant while participating in the study, she should inform study staff and her primary care physician immediately. Site staff will immediately report any pregnancy to the Investigator and procedures should be followed as outlined in Section 8.9. Participants found to be pregnant will not use any further study product but every attempt will be made by study staff to follow the pregnancy for the outcome. In addition, women who are breast-feeding are not to be enrolled in to the study.

7. STUDY PROCEDURES AND EVALUATIONS

This is a double-blind, placebo controlled, randomized, dose-ranging study in women diagnosed with BV according to the Amsel’s criteria and a Nugent score of ≥4.

After an approximate maximum 1 week screening period, participants will be randomized to receive 0.5%, 1% or 3% SPL7013 Gel or HEC placebo gel at a dose of 5g daily (at night) administered vaginally for 7 consecutive days.

Participants will be assessed for BV (both by Amsel’s criteria and by Nugent score) at each visit.

Visits should be scheduled as close as possible to the stated times, with a visit window of +/- 2 days.

The study flow chart below (Figure 1) outlines the design of the study and Table 1 in Page i summarizes the study procedures to be followed for each study visit.

Starpharma Pty Ltd, Baker IDI Building, 75 Commercial Rd, Melbourne, Victoria 3004, Australia

7.1 Assessment Periods and Study Procedures

Figure 1: Study Flow Chart

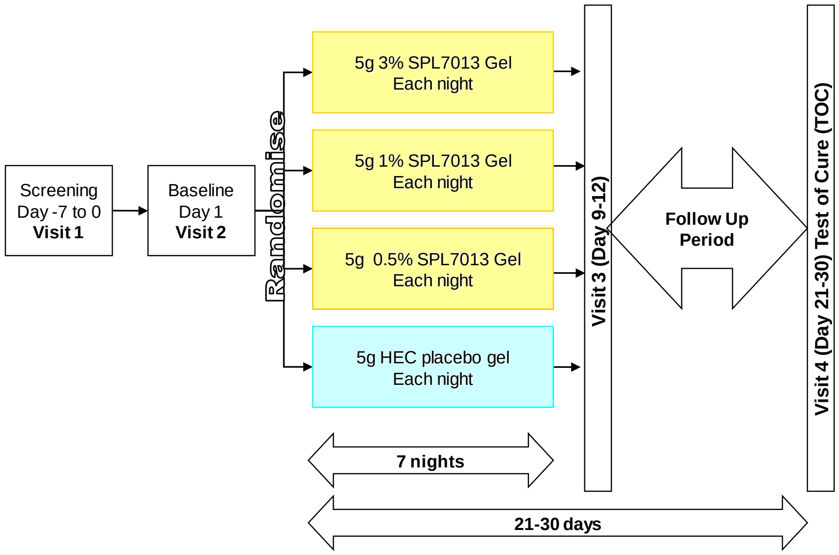

7.1.1 Visit 1 - Screening Evaluation

Screening procedures may be carried out up to 7 days prior to enrolment; however, as patients will require treatment as soon as possible it is preferable for the screening period to be as short as possible. Screening and enrolment/Baseline visits may be combined if it is possible to retrieve the laboratory results sufficiently quickly to confirm eligibility.

During the screening evaluation, each potential study participant will receive information on the study design and procedures, and risks and potential benefits of participation in the study. Each potential participant will have to read, sign and date the informed consent before any study procedures are performed.

The screening visit will include the following procedures and collection of the following information:

- Explanation of study requirements
- Sign and date informed consent
- Assign Screening ID
- Demographic data (including date of birth, race and ethnicity)
- Previous medical, surgical, menstrual and sexual history (including previous episodes of BV within the previous year and treatments, use of lubricants, douches, condoms, sex toys (sole use or shared), sexual practices in the previous 3 months and sexual orientation)
- Vital signs

- Physical exam only if indicated for a stable but pre-existing condition at the investigator's discretion
- STI screen (see Section 7.2.6).
- Pelvic exam with:
  - vaginal sampling for preparation of dry and wet mount slides for detection of trichomoniasis, clue cells, and/or yeast, and Gram stain (for Nugent scoring), semi-quantitative analysis (sub-set only), and storage sample (see Sections 7.2.3 and 7.2.6)
  - speculum examination of vagina and cervix
  - naked eye examination of vulva
  - assessment of vaginal pH
  - Pap smear if required
  - A local test for BV may be performed at the investigator's discretion to confirm diagnosis of BV prior to receipt of Nugent score.
- Assessment of BV according to the Amsel's Criteria
- Urine samples for pregnancy testing and urine dipstick for UTI (see Section 7.2.6)
- Completion of Symptom Questionnaire (see Section 7.2.7)
- Review of prior and concomitant medications
- Review of inclusion/exclusion criteria
- Review of study procedures and restrictions
- Demonstration of study product use
- Provide laboratory results with referral according to local practice when necessary

Participants who meet all the inclusion/exclusion criteria will be eligible for enrolment. If the time period for generation of the Nugent score is unacceptable to the investigator, the participant may be enrolled before confirmation of a score of 4 or more if a local test for BV returns a positive result. If the screening Nugent score is subsequently found to be less than 4, the participant must be withdrawn.

7.1.2 Baseline/Enrolment (Visit 2, Day 1)

Participants deemed to be eligible after the screening assessments (Nugent score may be outstanding as above if a local test for BV returns a positive result) will return for the Baseline/Enrolment visit, which should be scheduled after complete cessation of menses. This visit will include the following procedures and collection of the following information:

- Review of protocol compliance
- Review of the inclusion/exclusion criteria (see Section 5.1)
- Review of AEs and concomitant medications (see Sections 7.3.5 and 8). AEs occurring prior to administration of first dose of study product should be entered in to the Screening Signs and Symptoms section of the CRF.
- Pelvic examination including vaginal swab for Gram stain, semi-quantitative analysis (sub-set only) and storage sample (see Sections 7.2.3 and 7.2.6)

- Assessment of BV according to the Amsel's Criteria
- Urine samples for pregnancy testing and urine dipstick for UTI (see Section 7.2.6)
- Completion of Symptom Questionnaire (see Section 7.2.7)
- Issue diary card and provide instructions for completion
- Re-iterate study procedures and restrictions
- Confirmation of technique for study product use
- Randomization (see Section 6.4)
- Assign PIN
- Dispense study product

Each participant is then provided with the date of the next scheduled visit, and reminded to administer study product later that day just before bed and continue dosing for the remaining 6 days as directed.

7.1.3 Visit 3 (Day 9-12)

Visit 3 will be scheduled for Day 9-12, after completion of treatment. This visit should be scheduled prior to the anticipated date of onset of menses. If menses is due to occur or actually occurs on Day 9 then the visit may be delayed until complete cessation of bleeding.

This visit will include the following procedures:

- Review of protocol compliance
- Review diary card
- Review of AEs and concomitant medications (see Sections 7.3.5 and 8)
- Completion of Symptom Questionnaire (see Section 7.2.7)
- Completion of Acceptability Questionnaire [TSQM] (see Section 7.2.8)
- Pelvic examination including vaginal swab for Gram stain, semi-quantitative analysis (sub-set only) and storage sample (see Sections 7.2.3 and 7.2.6)
- Assessment of BV according to the Amsel's Criteria
- Issue diary card for follow-up period and provide instructions for completion
- Collect used and unused applicators

Women who report no improvement in vaginal discharge associated with BV who wish to receive further treatment will be offered rescue therapy according to Section 6.8. These study participants will be considered "Treatment Non-Responders", and will not attend the TOC visit. Instead, these participants will receive a telephone call at the time that the TOC would be scheduled to assess response to rescue medication and any adverse effects (*e.g.* candidiasis). Participants will be referred to their primary care physician for any ongoing AEs as appropriate. Any ongoing SAEs should be followed until resolution.

Each participant is then provided with the date of the last scheduled visit, the Follow-Up visit.

7.1.4 Visit 4 (Day 21-30) – Test of Cure (TOC)

Visit 4 (TOC) will be scheduled for Day 21-30, and will include the following procedures:

- Review of protocol compliance
- Review diary card
- Review of AEs and concomitant medications (see Sections 7.3.5 and 8). Participant will be referred to her primary care physician for any ongoing AEs as appropriate. Any ongoing SAEs should be followed until resolution or considered as stable.
- Completion of Symptom Questionnaire
- Pelvic examination including vaginal swab for Gram stain, semi-quantitative analysis (subset only), and storage sample (see Sections 7.2.3 and 7.2.6)
- Assessment of BV according to the Amsel's Criteria. Women who suffer a recurrence of BV symptoms who wish to receive further treatment will be provided with treatment for her BV according to local practice (see Section 6.8). Any rescue therapy will be documented in the participant's CRF.
- Pregnancy testing (see Section 7.2.6)

7.1.5 Interim Visits and Contact

Interim contacts and visits between regularly scheduled follow-up visits may be performed at participant request or as deemed necessary by the Investigator at any time during the study. All such visits will be documented in participant's study records and any AEs reported during such visits will be documented in the Case Report Form (CRF).

7.1.6 Withdrawal Visit

Any participant who prematurely withdraws from the study, for whatever reason, will be asked to attend a Withdrawal Visit as soon as possible and ideally within 2 weeks of finishing study treatment administration. This includes women who enroll in to the study with a positive local test for BV but without a confirmed Nugent score of 4 or more, where the Nugent score is subsequently determined to be less than 4, and are required to withdraw. Women who require rescue therapy are not considered withdrawals unless they do not complete the TOC visit; they are classified as "Treatment Non-Responders". Follow-up procedures for female participants who become pregnant during the study are outlined in Section 7.1.7. The Withdrawal Visit will include the following procedures:

- Review of protocol compliance
- Review diary card
- Review of AEs and concomitant medications (see Sections (see Sections 7.3.5 and 8). Participant will be referred to her primary care physician for any ongoing AEs as appropriate. Any ongoing SAEs should be followed until resolution or considered as stable
- Pelvic examination including vaginal swab for Gram stain, semi-quantitative analysis (subset only) and storage sample (see Sections 7.2.3 and 7.2.6))
- Assessment of BV according to the Amsel's Criteria

Starpharma Pty Ltd, Baker IDI Building, 75 Commercial Rd, Melbourne, Victoria 3004, Australia

- Pregnancy testing (see Section 7.2.6)
- Completion of Symptom Questionnaire (see Section 7.2.7)
- Completion of Acceptability Questionnaire [TSQM] (see Section 7.2.8)

Women in whom the BV has been found to recur at this Withdrawal Visit will receive rescue medication for their BV according to local practice as outlined in Section 6.8. Any rescue therapy will be documented in the participant's CRF.

7.1.7 Follow up Procedures for Female Participants Who Become Pregnant

Female participants who become pregnant during the study will be withdrawn from the study and undertake as much as possible of the Withdrawal Visit as outlined above without the pelvic examination (unless clinically indicated). Site staff will make every effort to remain in contact with the study participant until the birth in order to determine the status of both mother and child.

Pregnancies should be assessed and reported as outlined in Section 8.9.

7.2 Observation and Measurements: Assessment of Safety

7.2.1 Demographics and Medical, Surgical, Sexual, Menstrual History

Each study participant's demographics and medical, surgical, sexual and menstrual history will be taken from hospital records and, if applicable, clinic notes. During Screening, medical, surgical, sexual and menstrual history will be obtained from a physical examination and interview of the participant to ensure participants are eligible for the study. All relevant medical conditions identified will be recorded in the Medical History section of the CRF.

7.2.1.1 Demographics

Study participants date of birth, race and ethnicity will be collected. Additionally it will be noted whether the participant smokes, her employment status and whether she had at least 14 years' of education.

7.2.1.2 Medical and Surgical History

Relevant medical and surgical history will be collected focusing on those practices that may affect BV and including: any ongoing medical conditions or relevant conditions in the previous 5 years; surgical procedures in the previous 5 years or any gynecological surgery; previous pregnancy(ies); current method of contraception; history of BV over the previous year and treatments received for each episode of BV.

7.2.1.3 Sexual History

Each participant will be provided with a questionnaire to complete regarding her sexual history. This questionnaire will collect the following information: Sexual orientation; number of sexual partners; sexual practices in the previous 3 months; and use of lubricants, douches and sex toys.

7.2.1.4 Menstrual History

Details of each participant's menstrual history will be collected, including date of onset of last menses, approximate length of menstrual cycle, whether menstrual cycle is regular or irregular (women with irregular menstrual cycle are excluded from the study), and whether the participant has any menstrual abnormalities (*e.g.* metrorrhagia, menorrhagia, dysmenorrhoea etc).

7.2.2 Vital Signs / Physical Examination

Each participant will undergo an assessment of vital signs (pulse, systolic/diastolic blood-pressure and oral temperature) at screening. A physical examination will only be performed at the investigator's discretion at screening to assess a stable but pre-existing condition, and thereafter during the study due to the emergence of an AE.

7.2.3 Pelvic Examination

Each participant will undergo a pelvic exam at each study visit. The same investigator is to perform the pelvic examinations at Baseline, Visit 3 and Visit 4 for an individual participant in order to maintain consistency of reporting of genital AEs. All conditions identified at Screening or Baseline (prior to product administration) will be recorded in the Medical History or Screening Signs and Symptoms sections of the CRF, and all conditions first identified at subsequent visits post-product use including the follow-up visit (*i.e.* not identified at screening or Baseline) will be recorded in the AE section of the CRF.

The pelvic examination includes the following procedures:

- Naked eye examination of external genitalia
- Speculum examination of vagina and cervix
- Wet mount vaginal smear for assessment of yeast and BV and trichomoniasis
- Dry mount vaginal smear for Gram stain and Nugent Scoring
- Samples for semi-quantitative vaginal culture (at Baseline and Visits 2 and 3 in sub-set only)
- Vaginal sample for processing and storage
- Collection of pH sample from the vaginal wall
- Palpation of pelvis only required at investigator's discretion if symptoms of abdominal pain or abnormal bleeding reported.

During examination of vagina, cervix and vulva the investigator will determine whether any signs or symptoms of genital irritation are present, as defined in Section 8.3. These will be actively marked as present or absent in the CRF.

Any vaginal discharge observed during pelvic exam and considered associated with leakage of study product will be identified as such in the CRF. Discharge not associated with leakage of

product will be accompanied by a description and an associated condition (if applicable) (*e.g.* white curdy vaginal discharge, Candida-related *etc.*)

See Table 1 for a full outline of all study procedures.

7.2.4 Adverse Events

The Investigator and designated study personnel will monitor each participant for AEs during the study. All AEs reported between consent and final follow-up will be recorded in the CRF. All AEs reported before administration of study product will be entered in to the Medical History sections of the CRF, and all other AEs reported in the AE section of the CRF. All SAEs will be captured in the AE section of the CRF from time of consent. All AEs deemed to be signs of genital irritation (as identified in Section 8.3) will be designated as such in the CRF. In addition, abnormal vaginal discharge considered to be leakage of the gel will be designated as such in the CRF and will not be considered to be an AE. The investigator or designee will ask the participant's non-leading questions in an effort to detect AEs. Examples of these are:

"How are you feeling?"
or
"Since you were last asked, have you felt unwell or different from usual?"

In addition, participants should be encouraged to spontaneously report any unusual feelings or sensations at the study visits and between visits on the study diary card. See Section 8 for full details on adverse experience reporting.

7.2.5 Normal Vaginal Discharge

A normal physiological vaginal discharge is a mix of secretions from the cervical mucus glands and desquamation of vaginal epithelial cells. It is often described as being colorless and white or milky in appearance, drying to a yellow on underwear.

The color, consistency, and associated odor for an individual woman may vary at different stages of the menstrual cycle and can also be affected by method of contraception, and other factors. It is acidic, with a normal pH of 4.5 and associated with a predominant flora of lactobacilli.

Because of the wide individual and population variability in the appearances of normal vaginal discharge, in clinical practice it is often the patient perception of a change from her expected pattern in amount, consistency, associated odor, or development of other symptoms that will determine whether vaginal discharge is normal or abnormal.

7.2.6 Laboratory Testing

7.2.6.1 STI Screen

Tests for the following STIs will be conducted at the Screening visit and if clinically indicated during the study:

- Chlamydia – according to local practice
- Gonorrhea – according to local practice
- Trichomoniasis – by wet mount vaginal smear or according to local practice

Any participant found to be positive for an STI requiring treatment will not be permitted to enter the study until the infection has completely resolved (confirmed by re-testing) and treatment has been completed for at least 14 days prior to enrolment. Participants diagnosed during the study will be required to withdraw from the study and all details of the diagnosis and treatment must be recorded in the CRF.

Full details on all assays are outlined in the Study Procedures Manual.

7.2.6.2 Urinalysis

Conducted at Screening and each visit:

- Urine dipstick (with microscopy if required) to determine abnormalities suggestive of bacterial UTI
  Note: The presence of leucocytes, proteinuria, hematuria and a positive nitrite test is indicative of UTI. The presence of trace proteinuria alone should not be sufficient for a presumptive diagnosis of UTI.

7.2.6.3 Assessment of BV

During the pelvic exam at each visit, a wet mount vaginal smear will be performed and the pH of the lateral vaginal wall determined to assess the presence of BV. A dry mount vaginal smear will also be performed for Gram stain and Nugent Symptom Scoring.

A diagnosis of BV will be determined according to the following criteria:

- At screening all of the following symptoms from the Amsel's Criteria[15] will need to be present to indicate a diagnosis of BV: presence of white to grey homogenous discharge; positive whiff test (amine odor with addition of potassium hydroxide (KOH)); pH greater than 4.5; presence of clue cells with a decrease in lactobacilli morphotypes, and increase in non-lactobacilli morphotypes. Thereafter a diagnosis of BV will be indicated by the presence of three out of the four criteria above.
- In addition, the Gram stained smear will be scored according to the Nugent's method[16] to determine the status of the vaginal flora. A score of 0-3 indicates normal vaginal flora, a score of 4-6 indicates intermediate flora, and a score of 7-10 indicates a microbiological

diagnosis of BV. At screening, a score of 4 or more is required to be eligible for entry in to the study.

As part of the diagnostic criteria for entry in to the study, a Nugent score may be determined locally or centrally. In the event that the time taken to generate a Nugent score is unacceptable to the investigator, a local test for BV may be conducted, and the participant may be enrolled if the test is positive without confirmation of the Nugent score. If the screening Nugent score is subsequently determined to be less than 4, the participant is to be withdrawn.

At the end of the study all Gram stained slides will be independently read by two microscopists at a central laboratory for independent verification of scores. In case of discordance, a further third microscopist will assess the disputed sides. All central microscopic readers will remain blinded to treatment assignment.

If there is a discrepancy between the locally and centrally read Nugent score, the locally read score will take precedence over the centrally read score in determining whether a participant is eligible to enter the study. The centrally read score will take precedence over the local score in determining study endpoints.

7.2.6.4 Assessment of Candidiasis

The wet mount vaginal smear obtained during each pelvic exam will also be assessed for the presence of *Candida* spores. If *Candida* spores are present with symptoms of candidiasis, then oral treatment may be administered with no requirement for withdrawal post-Baseline. Once a participant has been randomized, vaginal treatments may not be administered for the period between baseline (Visit 2) and the TOC visit (Visit 4) as these may compromise the study endpoints. If oral treatment cannot be administered for the candidiasis and treatment is required prior to the TOC visit (Visit 4), then the patient must be withdrawn from the study. However, if treatment is required prior to randomization to study product, study participants will need to first be treated before randomization, and will need to comply with the inclusion/exclusion criteria (*i.e.* cannot be randomized less than 14 days after last dose of medication).

All details of candidiasis and associated treatment are to be recorded in AE and Concomitant Medication sections of the CRF. Presence of *Candida* spores alone with no symptoms of candidiasis are not to be reported as an AE.

7.2.6.5 Pregnancy Test

A urine pregnancy test will be performed at each visit, except Visit 3 (Day 9-12).

7.2.6.6 Semi-Quantitative Analysis of Vaginal Flora

A semi-quantitative culture-based assessment of various organisms will be performed at the Baseline, end of treatment (Day 9-12) and TOC Visits for a subset of 24 women. These

organisms will include: *Lactobacillus* species (H_2O_2 producing and non H_2O_2 producing), *Gardnerella vaginalis*, *Escherichia coli*, *Staphylococcus aureus*, anaerobic gram-negative rods (*Bacterioides*, *Prevotella*, *Porphyromonas*), *Enterococcus* species, Group B Streptococcus, and Candida species.

7.2.6.7 Sample for Storage

A vaginal sample is taken using a flocked swab. This is rotated in 400µl phosphate buffered saline (PBS), and frozen initially at -20°C, before final storage at -80°C. This sample will be stored indefinitely and used in the event that further analysis is required of the participants' vaginal flora.

7.2.6.8 Handling and Processing of the Biological Specimens

The handling and processing of laboratory samples is described in detail in the Study Procedures Manual.

Each study site will adhere to standards of good laboratory practice (GLP) and local SOPs for proper collection, processing, labeling, transport, and storage of specimens to the laboratories.

7.2.6.9 Storage and Destruction of the Biological Specimens

All biological samples will be destroyed once processing and reporting is complete for all study participants.

7.2.7 Symptom Questionnaire

Both Investigator-assessed and participants' perceived symptoms (*i.e.* odor and/or discharge) will be monitored throughout the study via completion of a Symptom Questionnaire, which is to be completed at Screening, Baseline (Visit 2), Day 9-12 (Visit 3) and TOC (Visit 4). The questionnaire will be split in to two sections, the first section will be a questionnaire for participants to complete, and the second section will be an assessment of symptoms by the Investigator.

Participants will be asked to indicate whether or not they have abnormal discharge and/or odor, and also to rate these two symptoms from 1 to 5 (one being normal and 5 being heavy discharge / strong odor) and compare their symptoms to the previous visit (except Screening Visit or Screen/Baseline combined visit). They will also be asked details about the discharge and odor if any is present.

Investigators will characterize the participants' discharge in terms of color, odor, and consistency. They will also provide an overall assessment of vaginal discharge as "normal" or "abnormal" based on these three characteristics, and distinguishing between normal physiological characteristics for each participant and stage of menstrual cycle (see Section 7.2.5).

7.2.8 Acceptability Questionnaire

Acceptability of the product to study participants will be determined via the use of the "Treatment Satisfaction Questionnaire for Medication, version 1.4" Acceptability Questionnaire, which has been validated in the domains of effectiveness, convenience, side-effects and overall satisfaction of medication.[17] All participants will be asked to complete the Acceptability Questionnaire at the Day 9-12 Visit (Visit 3). Detailed procedures for completion of each questionnaire are outlined in the Study Procedures Manual.

7.2.9 Diary Cards

Diary cards will be employed throughout the study from Screening onwards for the study participant to record gel use (including reasons for not administering a dose), sexual activity (including details of lubricants, condoms, sex toys (sole use or shared), and type of sexual activity), signs and symptoms of genital irritation, other AEs, female menstrual history and any concomitant medication.

7.3 Study Restrictions

7.3.1 Dietary

There are no dietary restrictions during the study.

7.3.2 Confinement

Participants will only be confined at site for the duration of the study assessments outlined for each study visit. There will be no over-night confinement at the study site.

7.3.3 Physical Activity

There are no restrictions on physical activity throughout the study.

7.3.4 Sexual Activity

Although discouraged, there is no restriction on sexual activity throughout the study. All vaginal sexual activity (*i.e.* oral-vaginal, penile-vaginal or vaginal masturbation) is to be recorded in the diary card. Other details of sexual activity such as use of condoms, lubricants, sex toys (sole use or shared) or sexual activity with a new or existing partner are also to be recorded.

7.3.5 Concomitant Medication

There will be no restriction on concomitant medications administered during the study, with the exception of those outlined below:

To protect the integrity of the lower genital tract and reduce the possibility of AEs due to agents other than the study products, any spermicides, diaphragms, or contraceptive vaginal rings should not be used during this study.

Similarly, study participants requiring vaginal preparations for conditions other than their BV, such as anti-fungals, or study participants requiring antibiotics (by any route), may not be enrolled in to the study until at least 14 days after the last dose, and resolution of symptoms. Antibiotics by any route may not be administered during the on-study period, and if required will lead to cessation of study product, however participants should continue to undergo the remaining study visits. Symptoms of candidiasis occurring in study participants during the study should be treated with oral preparations where possible, if vaginally applied preparations are required, the participant will be required to withdraw from the study. All medication is to be recorded in the CRF. Participants will be required to withdraw from the study if they require treatment for an STI.

Participants will be discouraged from using additional vaginal lubricants; however, if these are used during the study, the participant will not be required to withdraw and their use is to be recorded in the concomitant medication section of the CRF.

There are no additional restrictions on concomitant medications administered during the study. However, all medications must be reported and recorded in the CRF (including prescribed and over-the-counter medications, all vitamins and herbal remedies and other traditional preparations) from the first Screening visit through to the Follow-Up visit. To avoid potential confounding of AE data (*e.g.* abrasions, changes in vaginal microflora, and allergic reactions to product components) that may be related to menstrual hygiene products (except tampons), participants will be asked to report if they have used any products and these are to be recorded in the concomitant medication section of the CRF. Use of the participant’s preferred brand of tampons is acceptable and is not expected to confound AE data. Alcohol and recreational or street drug use will be recorded in the participant’s study notes and CRF only if needed for interpretation/documentation of observed participant health status.

Generic or trade name should be recorded in the CRF for products with one active ingredient, or the trade name recorded for combination products, including dose, form and duration of treatment. If the concomitant medication is required as a treatment for an AE, then this must also be recorded in the CRF.

8. SAFETY REPORTING

The reporting and documentation of AEs and clinically significant laboratory abnormalities or other assessments is an essential component of all clinical studies. Therefore, it is important that all investigational staff understand the requirements and responsibilities outlined below. It is the responsibility of the Investigator to ensure that all AEs and other clinically significant findings that occur during the conduct of a clinical study are documented and reported accurately.

Adverse event(s) should be documented in terms of a medical diagnosis(ies) where possible, rather than signs and/or symptoms.

Starpharma Pty Ltd, Baker IDI Building, 75 Commercial Rd, Melbourne, Victoria 3004, Australia

8.1 Adverse Event Definitions

8.1.1 Adverse Event (AE)

At each evaluation the investigator will determine whether any AEs have occurred by asking a non-leading question and/or reviewing the diary card. Follow-up of any AEs that occur will be conducted according to standard local practice.

An AE includes any untoward medical occurrence in a study participant who uses the investigational product (whether it is the investigational product or the placebo) and which does not necessarily have a causal relationship to the study product (whether it is the investigational product or the placebo).

The investigator will make a judgment regarding whether or not, in their opinion, the AE was related to the test product. However, even if the investigator feels there is no relationship to the test product, the AE must be reported. If any clinical AEs have occurred they will be recorded on the AE report page of the CRF and their intensity will be graded.

AEs may include:

1. The significant worsening of a disease or symptoms of a disease following administration of investigational product/drug.
2. An intercurrent illness with an onset after use of the investigational product.
3. Exacerbation (*i.e.*, increase in frequency or intensity) of a pre-existing condition or event.

An AE does not include a/an:

1. Medical or surgical procedure: the condition that leads to the procedure is an AE.
2. Situations where an untoward medical occurrence has not occurred (e.g. hospitalization for cosmetic/elective surgery, social and/or convenience admissions).
3. Overdose of either study drug or concomitant medication that does not result in any signs or symptoms. If any signs or symptoms of an overdose present, then these will be recorded as an AE.

8.1.2 Serious Adverse Event (SAE)

An SAE is defined as any adverse drug experience occurring at any dose[1] that results in any of the following outcomes:

- Results in death[2]
- Is life-threatening[3]
- Requires in-patient hospitalization or prolongation of existing hospitalisation[4]
- Results in persistent or significant disability / incapacity
- Is a congenital anomaly / birth defect

[1] **Occurring at any dose**: Does not imply that the participant is using the study product at the time of the event. Use may have been interrupted temporarily prior to the onset of the SAE, but may have contributed to the event.

[2] **Death**: Death is an outcome of an SAE, and not an SAE in itself. All deaths must be reported for participants on study and for deaths occurring within 30 days of last study drug dose or within 30 days of last study evaluation, whichever is longer, to Starpharma Regulatory and QA Departments, who in turn will ensure immediate (within 24 hours) reporting to all appropriate regulatory bodies within the required timelines. The investigator should supply Starpharma and the IEC / IRB with any additional requested information as available (e.g. autopsy reports and terminal medical reports).

[3] **Life-threatening**: The term "life-threatening" in the definition of "serious" refers to an event in which the participant was at immediate risk of death at the time of the event. It does not refer to an event which hypothetically might have caused death if it were more serious.

[4] **Hospitalization**: This is defined as the participant being hospitalized overnight, or the participant's hospital stay being prolonged for at least an additional overnight stay. Hospital admissions for a pre-existing condition (e.g. elective surgery) or for normal disease management procedures (e.g. chemotherapy) will not be considered as serious. Complications that occur during hospitalizations are AEs. If a complication prolongs hospitalization, it is an SAE.

Important medical events that may not result in death, be life-threatening, or require hospitalization may be considered a serious adverse event (SAE) when, based upon appropriate medical judgment, they may jeopardize the study participant and may require medical or surgical intervention to prevent one of the outcomes listed in this definition. Examples of such medical events include allergic bronchospasm requiring intensive treatment in an emergency room or at home, blood dyscrasias or convulsions that do not result in inpatient hospitalization, or the development of drug dependency or drug abuse.

8.1.3 Unexpected Adverse Drug Reaction (UADR)

Unexpected Adverse Drug Reaction is defined as an adverse reaction to study drug that is not identified in nature, severity or frequency in the IB or that is not expected from the characteristics of study drug.

8.2 Reporting of an Adverse Event

8.2.1 Timeframe for Reporting Adverse Events

AE reporting will begin from first dose and will continue throughout the study until the TOC visit. AEs occurring during the screening phase of the study and before administration of study product are to be reported in the Screening Signs and Symptoms section of the CRF. AEs occurring thereafter (*i.e.* after first administration of study product) are to be reported in the AE reporting pages of the CRF.

8.2.2 Timeframe and Timelines for Reporting Serious Adverse Events

SAE reporting will begin from consent and will continue until 30 days post last dose of study medication.

ANY SERIOUS ADVERSE EVENT (INCLUDING DEATH) **DUE TO ANY CAUSE** WHICH OCCURS DURING THE COURSE OF THE INVESTIGATION, WHETHER OR NOT RELATED TO THE STUDY PRODUCT, **MUST BE REPORTED IMMEDIATELY** (within 24 hours of the investigator becoming aware of the event) TO STARPHARMA REGULATORY & QA DEPARTMENT AS FOLLOWS:

Reporting Contact: VP Development and Regulatory Affairs or Delegate
XXXXXXXXXXXXX
XXXXXXXXXXXXX
XXXXXXXXXXXXXXXXXXXXXXXXXXXXXXXXX

Telephone contact must be made with either Starpharma or study monitor in order to make an initial SAE report. Specific contact details and 24-hour contact details are available in the Study Procedures Manual.

The Investigator (or delegate) will be requested to complete Starpharma's SAE form including as much information regarding the event that is available at the time of the initial report. Investigators should not wait to receive additional information to fully document the event before notifying Starpharma of an SAE.

The Principal Investigator or Co-Investigator must review and sign-off each SAE report to confirm that they have reviewed the SAE and the details are correct; however, this sign-off must not delay the initial reporting of the SAE and may be undertaken after the initial report has been made.

Prompt notification is essential so that legal requirements and ethical obligations to the participants participating in the study can be met. A telephone report must be followed by a written report including copies of relevant medical records, autopsy reports and other relevant documents.

When reporting SAEs, the investigator should not include the name or address of the individual participant.

Minimum requirements for SAE reporting:

- Protocol number
- Center name/ID
- Reporting Investigator's/physician's name
- Participant initials, randomization number, age and sex

- Study drug dose and duration of treatment
- Condition treated (medical diagnosis, if known)
- Nature and severity of the SAE
- Relationship of SAE to Study drug
- Potentially confounding factors/concomitant medication*
- Action taken/outcome*

* Where action was taken or concomitant medication was administered, ensure that corresponding CRF pages have been completed.

The investigator must also:

- Report all SAEs to the reviewing IEC / IRB within the time-line specified by the reviewing body;
- Submit follow-up reports to Starpharma until the SAE has resolved or, in the case of permanent impairment, until the SAE stabilizes.

If the SAE is study drug related and unexpected, the SAE will be reported by Starpharma to the regulatory authority(ies) within required timeframes (i.e. within 7 (fatal or life-threatening) or 15 (non-fatal or not life-threatening) calendar days of the event occurring and/or becoming aware of the event).

8.3 Recording an Adverse Event

- **Adverse events:** All adverse events are to be recorded on the 'Adverse Event' pages within the participant's CRF.
- **Serious Adverse Events:** In addition to the 'Adverse Event' page, SAEs are to be recorded on Starpharma Serious Adverse Event reporting form and then a copy is inserted into the study participant's CRF.

At each clinical evaluation the Investigator/delegate will determine whether any AEs have occurred. The participant will be questioned in a non-leading way and no specific symptoms will be suggested. If known, the medical diagnosis of an AE should be recorded in preference to the listing of individual signs and symptoms.

All AEs deemed to be signs of genital irritation will be identified by the Investigator as such in the CRF by checking a check-box and additionally will be noted as either being reported by participant (either spontaneously or in the diary card) or detected at pelvic/genital exam. Signs and symptoms of genital irritation are defined as follows:

Symptoms:
Pelvic pain; vaginal and vulvar pain; tenderness; vulval itching, edema, erythema, lesions (vesicles, blisters, erosions, ulcers), or rash; vaginal itching, dryness, excessive discharge; dysuria; vulvovaginitis; or dyspareunia.

Signs:
Vulval edema, erythema, lesions (vesicles, blisters, erosions, ulcers), or rash; vaginal edema, erythema, excessive discharge or lesions; cervical edema, erythema, discharge, or lesions; vulvovaginitis; or cervicitis.

The Investigator will make a judgment regarding whether or not, in his/her opinion the study product had any possible causal relationship to the AE. The Investigator will evaluate any changes in laboratory values, and make a determination as to whether or not the change is clinically important, and whether or not the changes were related to the study drug. However, even if the Investigator feels there is no relationship to the study drug, the AE must be recorded in the CRF.

Every AE must be assessed and the CRF entry reviewed and confirmed by the Principal Investigator or Co- or Sub-Investigator. This confirmation is indicated by an Investigator sign-off in the CRF.

If any AEs are present when a participant completes the study or when a participant is withdrawn from the study, the Investigator/delegate should make every effort to follow-up the participant until the AE has resolved or stabilized. All follow-up information (and attempted follow-up contacts) should be documented in the participant's medical records.

The severity of an AE experienced by the participant is graded in the first instance according to the Division of AIDS Female Genital Grading Table for Use in Microbicide Studies (Version 1.0 (Addendum 1) November 2007). Events not listed in the Female Genital Grading Table will be graded according to The Division of AIDS Table for Grading the Severity of Adult and Pediatric Adverse Events (Version 1.0, December 2004).

If an AE changes in severity, it should be a single entry in the CRF, and assigned the highest severity experienced.

Those AEs not covered within either of the above toxicity tables will be graded as follows:

Grade 0: None	Not an AE
Grade 1: Mild	An AE that causes no or minimal interference with usual social and functional activities.
Grade 2: Moderate	An AE that causes greater than minimal interference with usual social and functional activities
Grade 3: Severe	An AE that causes inability to perform usual social and functional activities.
Grade 4: Life Threatening	An AE that causes inability to perform basic self-care functions or medical or operative intervention is indicated to prevent permanent impairment, persistent disability or death.

It should be noted that an AE that is considered to be “severe” may not necessarily be considered to be “serious” or of major medical significance.

The relationship to study product should be assessed using the following definitions:

Not Related	The adverse event is clearly explained by another cause not related to the study product.
Probably Not Related	A potential relationship between study product and the adverse event could exist (i.e. the possibility cannot be excluded), but the adverse event is most likely explained by causes other than the study agent.
Possibly Related	The adverse event and administration of study product are reasonably related in time, and the adverse event can be explained equally well by causes other than the study product.
Probably Related	The adverse event and use of study product are reasonably related in time, and the adverse event is more likely explained by study product than other causes.
Definitely Related	The adverse event and use of study product are related in time, and a direct association can be demonstrated.

It should be emphasized that ineffective treatment should not be considered as causally related in the context of AE reporting.

These criteria in addition to good clinical judgment should be used as a guide for determining the causal assessment.

The degree of certainty with which an AE is attributed to study product or alternative cause, (*e.g.* natural history of the underlying diseases, concomitant therapy, etc.) will be determined by how well the event can be understood in terms of:

- Known pharmacology of the product;
- Reaction of a similar nature previously observed with similar products;
- The event having often been reported in literature for similar products as product related;
- The event being related by time to product use/termination, product withdrawal, or reproduced on rechallenge.

8.4 Clinical Laboratory Abnormalities and Other Abnormal Assessments as AEs or SAEs

Laboratory abnormalities such as an abnormal urinalysis deemed not clinically significant and not considered an AE according to the DAIDS toxicity tables are usually not recorded as AEs or SAEs. However, all abnormal laboratory findings or other abnormal assessments (e.g. pelvic

Starpharma Pty Ltd, Baker IDI Building, 75 Commercial Rd, Melbourne, Victoria 3004, Australia

exam) that meet the definition (or cause signs and symptoms that meet the definition) of an AE (or SAE) must be reported as outlined above.

8.5 Toxicity Management

AEs and laboratory abnormalities will be graded as outlined in Section 8.3.

For the purpose of monitoring toxicities, Baseline is defined as the status or last value prior to the first use of study product. All management is based on changes from this initial status or value.

All grade 2 or higher laboratory abnormalities should be confirmed by repeat testing as soon as possible, preferably within 3 calendar days of receipt of results prior to dose interruption or discontinuation, unless such a delay is not consistent with good medical practice.

Study participants will be asked to contact the study site staff if they miss a dose of study medication due to an AE. Site staff will assess whether the participant should come in for an assessment, and if so, an ad hoc visit will be scheduled as soon as possible. In response to AEs reported by participants and/or observed upon exam by study staff, the study site investigator or designee will recommend either continuation or holding study product use consistent with the criteria in Section 16.3. Study product use also will be held or discontinued in the event of an SAE that is judged by the site Investigator or designee to have a potential causal relationship with study product.

Unless the participant withdraws consent, they will remain in the study to complete the safety evaluations (unless clinically contraindicated according to Table 1 and Section 16.3).

Participants requiring treatment for an STI will be withdrawn from the study. Participants who develop any other conditions requiring vaginal therapy (*e.g.* candidiasis) whilst on study where oral therapy cannot be used or antibiotics by any route, will cease study product, however they should continue with the remaining study visits.

8.6 Follow-Up of Adverse Events

Investigators must follow up participants with SAEs until the event has stabilized or resolved whichever is the latter. In the case of non-serious AEs, follow-up should occur until the participant completes the study. Records of the participant's progress should be maintained until the conclusion of the study and thereafter in a participant's medical history at the relevant investigational site. Details of the participant's progress must be submitted to the Starpharma and Study Monitor on request.

8.7 Regulatory Reporting Requirements

Starpharma have a legal responsibility to notify the local regulatory authority (ies), and other overseas agencies (if applicable) about the safety of the product/drug under clinical investigation.

Prompt notification of SAEs by the Investigator is essential so that legal obligations and ethical responsibilities towards the safety of other participants are met.

8.8 IEC/IRB Reporting Requirements

The time frame within which the Investigators must be notified of deaths, study drug related and/or unexpected SAEs is stipulated by the local regulatory authorities.

It is the Investigators' responsibility to comply with the requirements for IEC/IRB notification. Each Investigator will notify the IEC/IRB, by promptly forwarding the Safety Report completed by Starpharma or its agent, for all onward reportable SAEs.

8.9 Pregnancy

Participants who become pregnant during the study period (up to and including 30 days after the last use of investigational product) must not use any further investigational product, and are to be instructed that they immediately notify the Investigator.

Starpharma must be notified within 24 hours of the Investigator learning of the pregnancy. The Investigator will be asked to complete the SAE CRF without the 'criteria of seriousness' checked.

Participants will be withdrawn from the clinical study and the procedure for discontinuation of participants who becomes pregnant will be followed (see Section 7.1.7).

Whenever possible, a pregnancy in participants exposed to study product should be followed to term. Any premature terminations and the status of the mother and child after delivery should be reported to Starpharma. The Pregnancy Outcome and Questionnaire Form may be used by the Investigator to record details of the pregnancy outcome. The form includes provision to record the mothers' status during the pregnancy, relevant medical history, and details of the health of the baby(ies) on delivery.

If a congenital anomaly/birth defect occurs this must be reported as an SAE.

8.10 Safety Monitoring

The following AEs will be reported by the Investigator to the study team on an ongoing basis:

- Any signs and symptoms of genital irritation graded at Grade 2 or above
- Any other AEs considered to be potentially related to study product reported at Grade 3 or above
- All SAEs

If the Investigator and/or study team consider that an abnormally high number of signs and symptoms of genital irritation, other Grade 3 AEs, or SAEs are being reported that have a potential causality to study product, then a blinded review of the AE data will be conducted. This

blinded review will include the Investigator, Medical Expert and Starpharma. From this review, one or more of the following recommendations may be made:

- No action need be taken;
- A protocol amendment may be implemented;
- Additional external advice may be sought;
- An independent review of the unblinded data may be conducted;
- The study may be paused or halted;
- Other appropriate recommendations may be made.

Actions will be reported to the IRB and/or relevant regulatory authorities if necessary according to their requirements.

9. CLINICAL MANAGEMENT

9.1 Participant Completion

A participant will be deemed to have completed the study once all trial procedures have been conducted. Study participants who have a relapse of their BV during the follow-up phase of the study will still be considered completed participants, and will be offered rescue therapy as per local practice, after completing the Follow-Up Visit. Any AEs or SAEs still ongoing will be followed in accordance with Section 8.

9.2 Treatment Non-Responders

A participant will be considered to be a "Treatment Non-Responder" if she reports that the abnormal discharge associated with BV has not improved at Visit 3 and wishes to receive alternative therapy. These participants will not be required to attend the TOC Visit, and will be provided with rescue therapy as per local practice. These women will receive a telephone call at the time that the TOC visit would have been scheduled to assess response to rescue therapy and any adverse effects of the therapy (*e.g.* candidiasis).

9.3 Study Participant Withdrawal

The investigator should make every reasonable effort to keep each participant in the study, except where termination or withdrawal is for safety reasons. However, if the investigator removes a participant from the study, or a participant declines further study participation, a complete exit evaluation must be performed in the form of the Withdrawal Visit. Study participants who do not respond to treatment are not considered withdrawals, but "Treatment Non-Responders" and are handled as above. Should a participant decide to withdraw from the study, all efforts will be made to complete and report the reason(s) for withdrawal and observations as thoroughly as possible.

The investigator may cease study product and withdraw the participant, or the participant may withdraw herself from participation in the study at any time and for any reason. The following are considered justifiable reasons for subject withdrawal from the study:

- The need to take medication which may interfere with study measurements;
- Intolerable/unacceptable adverse experiences;
- Major violation or deviation of study protocol (see Section 12.9);
- Participant unwilling to proceed and/or consent is withdrawn;
- Participant is pregnant;
- Withdrawal from the study is, in the investigator's judgment, in the best interest of the participant;
- Participant unblinded to treatment assignment;
- Lost to Follow-up;
- Other (*e.g.* personal reasons).

9.4 Procedures for Handling Withdrawals

The Investigator (or delegate) must complete the reason for withdrawal and the date of withdrawal of a participant in the appropriate section of the participant's CRF. If more than one reason applies, then the main reason for withdrawal should be indicated.

In the event that a participant becomes pregnant at any time during the study period every attempt will be made to follow her to term (although study participant has the option to decline further follow-up), the outcome of the mother and infant documented as per Section 8.9.

9.5 Replacement of Withdrawn Participant

Any participants who discontinue the clinical study of their own volition or who are discontinued by the investigator may be replaced at the discretion of Starpharma, irrespective of whether they have been assigned a PIN and have received a dose. Replacement of withdrawn participants will only be performed if an on-study withdrawal rate of more than 15% is observed during the study and following 50% of planned recruitment.

9.6 Lost to Follow-Up

At the start of the study each study participant will state how they can be contacted during the study. These contact details will be recorded in the participant's address file. If a participant fails to appear for a scheduled visit, at least three attempts to contact them should be made over the subsequent 7 days. These attempts should be documented in the participant's study file. After these three attempts, no further efforts need be made to find them, but their file should remain open until study closeout.

If the participant does not return to the clinic before the study is closed, the CRF should be checked to indicate that the participant was lost to follow-up. The "loss to follow-up" designation cannot be made for any participant until the closing date of the study.

9.7 Premature Termination of Study

The study may be terminated prematurely by the Investigator or his/her designee, the Sponsor, government or regulatory authorities, or site IRB/IECs if:

- The number and/or severity of AEs justify discontinuation of the study;
- New data become available that raises concern about the safety of the investigational product, so that continuation might cause unacceptable risks to subjects.

In addition, Starpharma reserve the right to discontinue the trial prior to inclusion of the intended number of subjects, but intend only to exercise this right for valid scientific or administrative reasons.

After such a decision, the Investigator must contact all participating subjects within two weeks, and written notification must be sent to the Ethics Committee.

9.8 Notification of Primary Care Physician

If the study participant consents, her primary care physician will be notified of her inclusion in the study. If desired, the participant may be given an open letter for her to hand to her primary care physician during the follow-up stage of the study so that AEs may be reported.

10. STATISTICAL CONSIDERATIONS

10.1 Review of Study Design

The primary aim of this randomized, double-blind, dose-ranging study is to determine the efficacy and safety of three different strengths of SPL7013 Gel (0.5%, 1% and 3%) compared with HEC placebo gel, when administered each night for seven consecutive days in women with a diagnosis of BV. Efficacy will be determined in a number of different ways; as Clinical Cure, Nugent Cure, and Therapeutic Cure at the Test of Cure Visit (Day 21-30). Initial efficacy will be determined immediately post completion of the course of treatment by assessment of these parameters at Visit 2, on Day 9-12 and also at Baseline. Safety will be determined via assessment of signs and symptoms of genital irritation and other AEs.

10.2 Criteria for Evaluation of Study Objectives

10.2.1 Primary Endpoints

The endpoint for evaluation of primary study objective (Primary Endpoint) is as follows:

- "Clinical Cure" at the Test of Cure (TOC) visit as defined by no abnormal discharge, as described by the Amsel's criterion for vaginal discharge, and fulfilling no more than one of the other three criteria.

10.2.2 Secondary Endpoints

The endpoints for evaluation of secondary study objectives (Secondary Endpoints) are as follows:

- "Nugent Cure", "Therapeutic Resolution of BV" and "Therapeutic Cure" at the TOC visit
- "Clinical Cure", "Nugent Cure", "Therapeutic Resolution of BV" and "Therapeutic Cure" at Day 9-12 (Visit 3)
- Both Investigator-assessed and patient perceived resolution of symptoms compared to Baseline, as determined in the "Symptom Questionnaire"
- Genital AEs considered to be possibly, probably, definitely related to study product
- All other AEs
- Acceptability of study treatment as determined in the "Acceptability Questionnaire" (the TSQM).
- To assess the correlation of BV signs and symptoms (via Symptom Questionnaire) with clinical diagnosis of BV
- Semi-quantitative analysis of vaginal flora (sub-set only)

10.2.3 Definition of Signs and Symptoms of Genital Irritation

The following cluster of symptoms will be employed in determining the number of women with signs and symptoms of genital irritation, and will be actively solicited in the CRF and diary card:

Symptoms:
Pelvic pain; vaginal and vulvar pain; tenderness; vulval itching, edema, erythema, lesions (vesicles, blisters, erosions, ulcers), or rash; vaginal itching, dryness, excessive discharge; dysuria; vulvovaginitis; or dyspareunia.

Signs:
Vulval edema, erythema, lesions (vesicles, blisters, erosions, ulcers), or rash; vaginal edema, erythema, excessive discharge or lesions; cervical edema, erythema, discharge, or lesions; vulvovaginitis; or cervicitis.

Each AE considered to be a symptom or sign of genital irritation will be identified as such in the CRF, and additionally will be noted as either being reported by participant (either spontaneously or in the diary card) or detected at pelvic/genital exam.

10.2.4 Definition of Clinical, Nugent, Therapeutic Cure

10.2.4.1 Clinical Cure

Clinical Cure is defined as the resolution of BV according to Amsel's criteria (*i.e.* no abnormal discharge, as described by the Amsel's criterion for vaginal discharge, and fulfilling no more than one of the other three criteria, when all four were present at Baseline: presence of white to grey homogeneous discharge; positive whiff test (amine odor) with addition of potassium hydroxide (KOH); vaginal pH greater than 4.5; and presence of clue cells).

10.2.4.2 Nugent Cure

Nugent Cure is defined as the absence of BV and normalization of vaginal microflora according to the Nugent scoring criteria (*i.e.* a Nugent score of 3 or less when a score of 7 or more was determined at Baseline).

10.2.4.3 Therapeutic Cure

Therapeutic Cure is a composite endpoint defined as the absence of BV according to both the Amsel's and Nugent scoring criteria (*i.e.* no abnormal discharge fulfilling the description of the Amsel's criteria and presence of either one or none of the remaining three symptoms identified in the criteria and a Nugent score of 3 or less irrespective of status at Baseline).

10.2.4.4 Therapeutic Resolution of BV

Therapeutic Resolution of BV is a composite endpoint defined as "Clinical Cure" and a shift to intermediate or normal flora according to the Nugent scoring criteria (*i.e.* no abnormal vaginal

discharge fulfilling the description of the Amsel's criteria and presence of either one or none of the remaining three symptoms identified in the criteria *and* a Nugent score of 6 or less irrespective of status at Baseline).

10.2.5 Definition of "Treatment Non-Responders"

Women who report no improvement in the abnormal vaginal discharge associated with BV by Visit 3 (Day 9-12) and receive rescue therapy prior to the TOC Visit (Day 21-30) are considered to be "Treatment Non-Responders".

10.2.6 Description of Groups for Analysis

Analysis maybe performed on selected subgroups and presented for the primary and secondary efficacy endpoints where appropriate. Full details of any subgroups will be provided in the statistical analysis plan.

10.2.6.1 Description of Participant Completion

A participant is considered to have completed the study if they have administered at least 6 doses of study medication and attended all study visits.

10.2.6.2 Randomized Dataset

The randomized dataset will include all participants that meet the eligibility criteria and are randomized to double-blind investigational product. In determining eligibility, and in case of discrepancies, the Nugent score obtained at screening will take precedence over the score determined centrally.

10.2.6.3 Modified Intent to Treat Safety Dataset

The Modified Intent-to-treat (MITT) dataset will include all participants who are randomized to double-blind investigational product, administer at least one dose of study gel, and have at least one post baseline assessment of efficacy and did not receive antibiotics except as rescue medication (via any route) or vaginal antifungals. This dataset will be employed to determine all primary and secondary efficacy endpoints. Participants will be analyzed according to the treatment group in which they were randomized, irrespective of actual treatment received.

10.2.6.4 Safety Dataset

The Safety dataset will include all participants who are randomized in to the study and administer at least one dose of study gel. This dataset will be employed to determine all safety endpoints. Participants will be analyzed according to the actual study treatment received.

10.2.6.5 Per-Protocol (PP) Dataset

The Per-Protocol dataset is a subset of the Modified Intent-to-Treat dataset which includes all randomized participants who administered at least 6 doses of study product over 7 consecutive

days and who are considered sufficiently compliant with the protocol. All primary and secondary efficacy evaluations will be conducted in the Per-Protocol Dataset as supportive to the MITT analysis.

The definition of "sufficiently compliant" includes, but is not limited to:

- No violations of any study entry eligibility criteria;
- Received study treatment to which they were randomized and did not receive the wrong medication or incorrect dose at any time;
- ≥85% compliant to intended dose of Investigational Product;
- Did not receive any excluded concomitant medication prior to the time point evaluated;
- Treatment assignment was not unblinded to the subject, study site personnel, CRO, or Sponsor at any time during the study.

The criteria may be amended prior to unblinding of treatment assignment and analysis of data. Reasons for changes may include study conduct changes, protocol amendments, or changes in the appropriateness of the criteria, which may be assessed based on review of blinded data.

10.2.6.6 Microbiological Dataset

The microbiological dataset is a subset of the Intent-to-Treat dataset which includes participants that have at least one sample collected for semi-quantitative analysis of vaginal microflora.

10.3 Exclusion of a Study Participant from Statistical Analysis

Analysis and reporting of the study is to be conducted strictly in accordance with the Statistical Analysis Plan, and is to include all study participants fulfilling the definition of the datasets indicated above. Any decisions regarding the "evaluability of participants" is to be made in a blinded review of the data before database lock and agreement to exclude a particular participant's data from a dataset is to be clearly documented with a reason in the Study File and Final Study Report.

10.4 Sample Size Justification

Primary analysis will be a comparison of 0.5%, 1% and 3% SPL7013 Gel vs. HEC placebo gel with each treatment regimen. Assuming Clinical Cure rates of 50% and 15% for SPL7013 Gel and HEC placebo gel, respectively, a sample size of a minimum of 28 evaluable subjects per treatment arm will provide 80% power to detect a treatment difference with a final alpha significance level of 0.048. It is estimated that there will be a 15% drop-out rate which provides 33 patients to be randomized per treatment arm and 132 patients overall in the study.

The primary hypothesis will be:

Null Hyothesis (Ho): Clinical cure rate at TOC will be the same for both SPL7013 Gel and Placebo treated groups

Starpharma Pty Ltd, Baker IDI Building, 75 Commercial Rd, Melbourne, Victoria 3004, Australia

Alternate Hypothesis (Ha): Clinical cure rate at TOC will be different between SPL7013 Gel and Placebo treated groups

An interim analysis has been included in the sample size design using the using the O'Brien-Fleming method with the significance level calculated for the final analysis being 0.048.

10.5 Planned Interim Analysis

When 50% of patients have completed TOC at Day 9-12 visit, an interim analysis will be conducted to provide guidance in the decision of which dose groups to include in a forthcoming protocol based on preliminary efficacy results. The interim analysis will be carried out using the O'Brien-Fleming method with $\alpha=0.005$ for the interim analysis and $\alpha=0.048$ for the final analysis.

The interim analysis will be analyzed by an independent statistician not otherwise involved in the study. Full details of data to be analyzed and procedures for unblinding including dissemination of results will be presented in the statistical analysis plan.

10.6 Randomization and Blinding Procedures

Participants will receive either 0.5%, 1% or 3% SPL7013 gel or HEC placebo gel depending upon the randomization schedule.

Randomization of the allocation of each study product will be performed by an independent statistician employing a randomly permuted block design. Participants will be assigned a PIN upon randomization, and this number will link to the study product allocated.

The study is double-blind, *i.e.,* study staff and participants will be blinded to the random assignment of product to all study participants. All study products be supplied in identical packaging. Blinding will be maintained until all data are entered in to the study database, all study endpoint data and other data included in the final analysis have been cleaned and verified, and the data are ready for final analysis. This will be explained to participants as part of the study. The only person(s) to whom the study will be unblinded during its duration will be the independent statistician(s) preparing the randomization list.

There are no circumstances under which it is expected that unblinding will be necessary for the provision of medical treatment or to otherwise protect the safety of study participants. As described in Section 9.4 in the event that an Investigator is concerned that a participant might be put at undue risk by continuing product use, the Investigator may discontinue use for the participant concerned.

10.7 Safety Review

Safety review will occur throughout the study as outlined in Section 8.10.

10.8 Statistical and Analysis Plan

A final statistical analysis plan will be developed and approved by Starpharma prior to database lock. Statistical analyses will be performed after all participants have ended their participation in the study. Participants withdrawn prior to randomization will not be assessed for outcome variables or safety parameters. The following is a summary of the planned analyses; full details are captured in the statistical analysis plan. Any deviations to be made from this summary plan and planned analyses will be documented in the statistical analysis plan.

All hypotheses will be tested for statistical significance with two-tailed p-values unless otherwise noted. Results of all tests will be considered statistically significant if their p-value is less than or equal to 0.048 as per the O'Brien-Fleming α adjustment for the planned interim analysis.

Continuous variables will be summarized using descriptive statistics (N, mean, standard deviation, median, minimum and maximum), while categorical variables will be summarized as counts and percentages of participants in each category. Results will be presented by treatment group as appropriate.

Efficacy analyses will be presented using the MITT and per-protocol populations. For efficacy analyses, all missing values due to participant withdrawal and missed visits as well as values outside of analysis windows will be imputed using the last observation carried forward (LOCF) method as appropriate. For sensitivity analysis, efficacy results will also be presented with "as observed" results.

All baseline and demographic summaries will be based on the MITT dataset. No statistical comparisons between treatment groups will be made on demographic and baseline characteristics.

If there is a discrepancy between the locally and centrally read Nugent score, the locally read score will take precedence over the centrally read score in determining whether a participant is eligible to enter the study. The centrally read score will take precedence over the local score in determining study endpoints.

10.9 Analysis of Efficacy

The analysis of the primary efficacy variable will be performed using the MITT (as observed), MITT (LOCF) and Per-Protocol datasets.

The number of participants, percent of participants with Clinical Cure at TOC visit and exact 95% confidence limits will be displayed. The 95% confidence interval will be determined from the Poisson (incidence rate) distribution. The Clinical Cure rate will be compared between treatment

groups using Cochran–Mantel-Haenszel (CMH) test controlling for study center.

If the overall comparison across treatment groups is significant, pairwise comparisons of each SPL7013 treatment dose level with HEC placebo gel will be performed using the Chi-square test 95% confidence intervals for the difference in Clinical Cure rates between each SPL7013 concentration and HEC Placebo will be presented.

The analysis of the secondary efficacy variables that are based on categorical variables will be summarized using the same method as outlined for the primary efficacy variable.

Continuous secondary efficacy variables (*e.g.* questionnaires) will be compared for total score and change from prior visit between all treatment groups using an ANOVA model with treatment and study center as factors to test treatment effect.

Ordered variables will be compared between treatment groups and placebo using a non-parametric method where appropriate such as the Kruskal-Wallis test.

Patient reported outcomes will be considered as exploratory analyses. Symptom questionnaire responses at each visit will be presented as categorical and ordered variables. To assess the association between laboratory-verified diagnoses of BV (defined as Nugent score ≤3) and BV signs and symptoms, a univariate logistic regression will be performed.

Treatment Satisfaction Questionnaire for Medication (TSQM) transformed scores will be presented for each domain (effectiveness, side effects, convenience, and overall satisfaction) using summary statistics. The responses will be treated as continuous variables and analyzed using ANOVA to test for treatment effect.

Semi-quantitative analysis of vaginal microflora will be presented descriptively on a subset of participant's using the microbiological dataset. No statistical comparison will be performed on this data.

10.10 Analysis of Safety

All safety summaries will be presented using the safety dataset and comparing across all treatment groups unless otherwise noted.

The extent of exposure to study treatment will be quantified using the parameters total dose (mg), number of doses, and duration of exposure (days). Total dose and duration of dosing will be summarized using descriptive statistics while number of doses will be presented by counts and percentages. All drug exposure data will be listed and summarized by treatment group.

Percent compliance and the number of missed doses will be summarized using descriptive statistics for overall study and by treatment group. Compliance will also be presented according

to range categories and summarized by counts and percentages.

For AEs, the investigator's verbatim term of each AE will be mapped to system organ class and preferred term using the current version of MedDRA. Adverse events will be summarized by system organ class and preferred term; a participant will only be counted once per system organ class and once per preferred term within a treatment group. Participant counts and percentages and event counts will be presented for each treatment group by decreasing frequency for the following summaries; all treatment emergent adverse events (TEAEs), all SAEs, all TEAEs by maximum severity, all TEAEs by relationship to study treatment, all TEAEs related to study treatment.

Listings will also be presented by subject for all AEs as well as for SAEs, AEs leading to discontinuation from the study and AEs leading to discontinuation from study treatment. These listings will include treatment group and study period along with variables describing the nature, duration, and resolution of the event.

All AEs listed above will be presented separately by study period (on-treatment period or follow-up period) as well as overall for the study. Study period will be based on planned timing of visits (as outlined in Section 7) as opposed to whether particular participants were using study product or not. Adverse events will be split in to two groups: signs and symptoms of genital irritation considered to be possibly, probably and definitely related to study product (as defined in section 10.2.3 judged not to be due to pathogen or iatrogenic trauma); and all other AEs (non-genital). A summary of genital AEs will also be presented split out by AE grade as determined by the DAIDS toxicity grades. All AE summaries will be presented for both the safety and the Per-Protocol datasets.

All AEs determined to be signs or symptoms of genital irritation with a perceived potential causality to study product (*i.e.* assessed as Definitely, Probably, or Possibly Related) will be compared between treatment groups and exact 95% confidence limits will be displayed. The 95% confidence interval will be determined from the Poisson (incidence rate) distribution. The incidence of genital AEs will be compared across treatment groups using Cochran–Mantel-Haenszel (CMH) test controlling for study center.

A similar comparison will also be performed for incidence of reported signs of genital irritation irrespective of assigned causality.

All AEs will be reviewed by the Investigator and Sponsors at the end of the study in a blinded review to ensure that signs of genital irritation are correctly identified as part of the data-cleaning process.

The incidence of candidiasis and use of rescue therapy for BV relapse will be presented across all treatment groups.

11. HUMAN SUBJECTS PROTECTION

11.1 Regulatory Considerations

Starpharma or their agents will submit the appropriate documents to the local regulatory agencies and IEC/IRBs affiliated to each site and will await approval prior to study commencement.

This study will be conducted in accordance with the following guidelines and regulations:

- International Conference on Harmonisation (ICH) Guidelines for Good Clinical Practice
- The Declaration of Helsinki
- US Food and Drug Administration (FDA) Human Participant Protection Regulations (Title 21 Code of Federal Regulations, Parts 50, 54, 56 & 312)

11.2 Independent Ethics Committee (IEC)/Institutional Review Board (IRB)

Prior to the commencement of the clinical study, written IEC/IRB approval must be received by the Investigator.

The Investigator must submit the protocol, plus participant information and consent forms, for independent review by a recognized IEC/IRB pertinent to the study location.

The IEC/IRB should be constituted in accordance with local regulatory requirements.

Participant recruitment will not start until satisfactory evidence of ethical approval is given by the Investigator to Starpharma in writing. Starpharma require written approval that clearly identifies the study protocol by title, number, and version date. The study site must have copies of the IRB procedures and roster (or a statement of appropriate constitution of the IRB) in their regulatory file.

11.3 Interpretation of the Protocol / Protocol Amendments

With the exception of emergency situations, no changes or deviations in the conduct of the signed protocol will be permitted without the prior documented approval of Starpharma, Starpharma Medical Monitor/responsible physician(s) and the IEC/IRB. In the event of an emergency, the Investigator will institute any medical procedures deemed appropriate. However, all such procedures must be promptly reported to Starpharma, Starpharma Medical Monitor/responsible physician(s) and the IEC/IRB.

Administrative changes of the protocol are defined as minor corrections and/or clarifications that have no effect on the way the study is to be conducted. These administrative changes will be agreed upon by Starpharma and the investigator and will be documented in a memorandum. The IEC/IRB will be notified of administrative changes by the investigator.

Following approval of protocol amendments, an amended final protocol will be prepared.

11.4 Subject Informed Consent

Written informed consent will be obtained from all potential study participants prior to the initiation of any study-related procedures. In obtaining and documenting informed consent, the Investigator and his/her designee(s) will comply with applicable local and domestic regulatory requirements and will adhere to GCP and to the ethical principles that have their origin in the Declaration of Helsinki.

Prior to the beginning of the trial, site investigators will have the IRB/EC's written approval/favorable opinion of the protocol, informed consent forms, and any other study-related information to be provided to participants. This study does not plan to enroll children.

Participants will document their provision of informed consent by signing their informed consent forms. Participants who are illiterate will not be permitted to enter the study.

The informed consent process will give individuals all of the relevant information they need in order to decide whether to participate, or to continue participation, in this study. All study related materials distributed to participants including the informed consent forms will be available in English only. Potential participants will be permitted to ask questions and to exchange information freely with the study investigators. Only listed study investigators may obtain informed consent from potential study participants. The investigators will keep research participants fully informed of any new information that could affect their willingness to continue study participation.

11.5 Ethical Considerations

During the study screening process, participants will be tested for sexually transmitted diseases and managed according to local requirements. Persons found to be infected will be referred for treatment. Study staff will comply with all applicable local requirements to report communicable diseases to local health authorities. Participants will be made aware of all reporting requirements during the study informed consent process.

11.6 Confidentiality

Members of the study site staff are all trained in subject confidentiality. The log of study participant names and other protected health information will be kept in a locked area. All computer information about study volunteers will be kept on a computer with log-on passwords. Laboratory specimens are labeled with study numbers and date, and are delivered or shipped by study staff. The study sites' data management and clinical staff are the only personnel with access to the protected health information of study volunteer. All research records will be kept according to ethics committee, ICH and regulatory requirements (whichever is the longer) following closure of the study.

Starpharma will preserve the confidentiality of subjects taking part in this study. In the event of

names inadvertently appearing on study documentation, this information will not be processed.

Subject medical records pertaining to this study may be inspected/audited at any time by Starpharma employees or their duly authorized representatives, a regulatory authority or the IEC/IRB. All records accessed will be strictly confidential. Consent to participate in this study includes consent to these inspections/audits.

12. ADMINISTRATIVE ASPECTS

12.1 Clinical Trial Agreement

Prior to commencement of the study, the Investigators must sign a clinical trial agreement that will clearly delineate the responsibilities and obligations of the Investigator and Sponsors and will form the contractual basis under which the clinical trial will be conducted.

12.2 Study File

All associated study correspondence will be filed by the Investigator and will be available for inspection by the study monitor and/or appropriate representatives of Starpharma and/or regulatory authorities to determine that all documentation is present. It will be responsibility of the Investigator to provide adequate means for organization and filing of study documentation at the study centers.

12.3 Initiation of the Study

Prior to the commencement of the study, a designated representative of Starpharma will visit the investigational site to ensure adequacy of facilities and to discuss with the Investigator, and other personnel involved with the study, their responsibilities with regard to protocol adherence.

The investigational staff may not enroll any participants prior to receipt of written approval from the IEC by Starpharma, and completion of a formal meeting conducted by a Starpharma representative to initiate the study. This meeting will include an inventory of study supplies and a detailed review of the Protocol and CRFs.

12.4 Participant Reimbursement

Participants will be reimbursed according to the guidelines of the local IRB/IEC in order to compensate them for their inconvenience and time.

12.5 Participant Identification

All participants screened for a study will have their initials entered chronologically on the Participant Log at the initial visit. In the event that a participant is not included in the study, the reason is to be documented in the space provided on the Participant Log.

The eligible participants entering the Study will be assigned a Participant Identification Number

Starpharma Pty Ltd, Baker IDI Building, 75 Commercial Rd, Melbourne, Victoria 3004, Australia

(PIN) in sequential order. The Participant Identification Number of the participant will be entered on all pages of the participating participants' CRFs.

12.6 Confidential Follow-up

The Investigator will be responsible for retaining sufficient information about each participant (*e.g.* name, address, phone number, and identity in the study) so that regulatory agencies or Starpharma may access this information should the need to do so arise. These records should be retained in a confidential manner, as legally mandated according to local requirements.

12.7 Recording of Data

The investigator should maintain the individual participant files separate to the CRFs. The files should include visit dates of the participant, records of vital signs, medical history or examinations administered, laboratory results, concomitant treatments. Any AE encountered and other notes as appropriate. This constitutes 'source data'. All entries on the CRFs must be backed up by source data, unless agreed that the CRF will constitute source data.

The CRFs must be kept in order and up-to-date so that they always reflect the latest observations on the participants enrolled in the study.

Each participant's file should have attached to it the original signed Informed Consent. When the study treatment is completed, the Informed Consent should be kept on file with a copy of the completed CRF in the appropriate file folder provided, or a note made indicating where the records can be located. All records should be kept in conformance with applicable national laws and regulations.

All original laboratory reports should be available for review in each participant's file. It is important that the original reports are available for review because of the possibility of inaccuracies or errors in transcribing data from original records to the CRF.

CRFs must be completed legibly for each participant enrolled in the study and signed-off by the investigator. This should be done as soon as possible after completion of therapy. The Sponsor or designee will review and collect the CRF.

12.8 Monitoring of the Study

The Investigator will permit Starpharma and their agents to monitor the study as frequently as Starpharma deem necessary to determine that data recording and protocol adherence are satisfactory. A designated representative of Starpharma in the form of a Study Monitor will collect CRFs and to verify participant data on the CRFs for analysis.

The Investigator will allow Starpharma and their agents reasonable access to the CRF and related source documents for monitoring purposes as frequently as the sponsor deems necessary. This

includes tests performed as a requirement of participation in this study and may also include other medical records required to confirm information contained in the CRF such as past history and secondary diagnoses.

At each participant visit, the Investigator or delegate should record all data generated since the last visit on the CRF. The investigator and his/her staff will be expected to co-operate with the monitor to assist in providing any missing information.

The Study Monitor will require access to the Investigator's study file to ensure completeness of all study-related documentation. The Study Monitor will provide the investigator with adequate means for organization and filing of study documentation at the study center.

The date the Study Monitor visits the investigational site will be recorded in the site visit log. During monitoring visits, the study site co-coordinator and Investigator should be available, the source documentation will be accessible and a suitable environment will be provided for the Study Monitor to review study related documentation.

The key purposes for monitoring visits by the Study Monitor include the following:

i. Helping to resolve any problems.
ii. Examining all study documentation for completion, adherence to the protocol and possible AEs.
iii. Discussing inconsistencies or missing data.
iv. Ensuring all study materials are correctly stored and dispensed.
v. Verification of study data with source documents.
vi. Checking fulfillment of the obligations of the Investigator.
vii. Reviewing consent forms and date of consent.
viii. Inspecting study drug (storage, labeling and documentation).

The investigator will provide Starpharma or their designated representative with the completed and signed CRFs at the end of the study period planned for each participant (*i.e.* as defined by the protocol, and within 2 weeks of the last participant completing the study).

12.9 Protocol Deviations

Deviations from the protocol should not be made other than as part of a protocol amendment agreed with Starpharma, except where necessary to eliminate an immediate hazard to study participants or when the change(s) involves only logistical or administrative aspects of the study.

All protocol deviations must be noted and explained in the Investigator's file. Participants may only be withdrawn from the study or statistical analyses during a blinded review of the data.

Protocol deviations can be divided into two categories as follows:

Minor Protocol Deviations

- all instances where the protocol specified instructions have not been followed during the on-study period.

  For example:
  - a visit occurs outside the acceptable window period;
  - the required number of applications of study product have not been reached;
  - concomitant vaginal products are used.

Major Protocol Deviations (also known as "Protocol Violations")

- when the participant did not meet all inclusion and/or exclusion criteria prior to entry into the study;
- if additional procedures are performed that are not specified in the protocol.

All major protocol deviations are to be reported to Starpharma, and the IRB if required.

12.10 Data Quality Control

Throughout the Study, the data will be monitored and the CRFs checked against the participant's medical record for completeness and accuracy. This will be performed by Starpharma or its legally contracted agents.

Following completion and collection of the CRFs, the data will be checked manually, entered into a database and electronically checked for consistency and range. Queries will be generated for spurious data and clarification sought from the responsible Investigator or delegate at the Study site.

These data queries must be resolved in a timely manner by the Study site.

12.11 Quality Assurance Audit/Inspection

The Study may be participant to an audit by an authorized representative of Starpharma and/or an authorized Regulatory Authority (*e.g.* FDA, MHRA).

Regulatory authorities may request access to all study documentation, including source documents for inspection and copying, in keeping with local regulations. Starpharma will immediately notify the Investigator of an upcoming audit/inspection.

In the event of an audit, all pertinent study-related documentation must be made available. If an audit or inspection occurs, the Investigator will permit the auditor/inspector direct access to all relevant documents and allocate his/her time as well as the time of relevant staff to discuss the findings and any relevant issues.

12.12 Study and Site Closure

Starpharma reserves the right to prematurely discontinue or suspend the study either at a particular site or at all sites at any time and for any reason. If such action is taken, Starpharma will discuss this with the Investigator(s) at that time and notify the Investigator(s) in writing. If the study is suspended or terminated for safety reasons all Investigators conducting the study will be immediately notified of the action as well as the reason for it, as will the relevant regulatory agencies. The Investigator will advise the IEC/IRB overseeing the study at their site.

Upon closure of the study (whether at the expected conclusion or prematurely), the following activities will be performed by the Sponsor in conjunction with the Investigator:

1. Return of all study data
2. Data clarification and resolution of queries
3. Study product/drug accountability, reconciliation and final disposition
4. Review of site study records for completeness
5. Return of randomization codes
6. Shipment of all relevant samples to the central laboratory

12.13 Record Retention

All study documents, including the protocol and CRFs, are the confidential property of Starpharma and should be regarded as such. Unused CRFs must be returned to Starpharma or destroyed at the end of the study. Completed CRFs will be returned to Starpharma during the study by a method agreed by the study monitor. A study document binder will be provided by Starpharma for all required study documents. A checklist of all records to be retained by the Investigator will be provided by Starpharma.

Following completion of the study the Investigator will retain copies of the approved protocol, completed CRFs, informed consent documents, relevant source documents, and all other supporting documentation related to the project in accordance with the applicable ethics committee, ICH and regulatory requirements (whichever is the longer). Documents must be retained for a minimum of 15 years from the date of termination of the study or for at least 2 years after the last approval of a marketing application in an ICH region, or at least 2 years after the formal discontinuation of the clinical development of an investigational product.

In the event that the Investigator retires or relocates, custody of the records may be transferred to another suitable person who will accept responsibility for the records. Notice of such transfer should be given to Starpharma in writing.

12.14 Study Report

A complete study report and its results shall be written on completion of the study and will include any conclusions drawn with respect to the safety and efficacy of the study product (refer to ICH Topic E3 - Note for Guidance on Structure and Content of Clinical Study Reports (CPMP/ICH/137/95)).

Starpharma may also request the preparation of an interim report for submission to a regulatory agency. Starpharma or its agent will write the report in consultation with a nominated Investigator (or nominee). It is the expectation of Starpharma that the Investigator will sign the final study report.

Progress reports will be provided to the FDA, MHRA, IRB/IEC and all other relevant regulatory bodies in accordance with their requirements.

13. INDEMNITY AND COMPENSATION

13.1 Compensation

Starpharma will provide compensation to participants for any injury suffered as a result of participation in the study in accordance with the ABPI guidelines, as modified by Medicines Australia for compensation for injury resulting from participating in a company-sponsored research project. A copy of the guidelines is available to participants from the research staff on request.

13.2 Indemnity

Starpharma will indemnify persons involved in the study under the terms of the Indemnity set out in the Clinical Trial Agreements. The indemnity covers the Investigator, the Hospital and the Ethics Committee for any liabilities arising out of damage to a patient/healthy volunteer participating in the Study.

Exclusions
The Indemnity covers reasonable exclusions. For example it will not operate if the damage arises from a breach of the Protocol or the Clinical Trial Agreement. It will also not operate to the extent that the damage arises from the fault, negligence or malpractice of a person indemnified.

Notification
It is imperative that the persons indemnified promptly notify Starpharma of any claim, intended claim or any adverse reaction or incident, which could give rise to a claim.

13.3 Insurance

Starpharma has taken out insurance to cover its obligations under Indemnity and compensation guidelines for injury to patients involved in the Study.

14. SPONSOR RESPONSIBILITIES

In addition to preparing the study protocol Starpharma or their agents will also be responsible for the conduct of the activities listed below.

14.1 Funding

Starpharma will fund the study as outlined in the Clinical Trial Agreements. All direct costs associated with the conduct of the study and laboratory investigations will be paid for by Starpharma as outlined in the Clinical Trial Agreements.

14.2 Supply of Study Materials and Study Documentation

Starpharma or their agents will supply the study materials including CRFs, participant diary cards and other associated documentation required for the study.

14.3 Compliance with Regulatory Requirements

Starpharma will ensure that the Investigator is conducting the study in accordance with the local and international regulatory requirements as stipulated in the protocol.

14.4 Transfer of Sponsor Obligations

Transfer of Sponsor Obligations may occur for certain activities such as monitoring and data management.

15. USE OF DATA AND PUBLICATIONS

Starpharma may disclose data derived from the study to other Investigators and national or foreign regulatory authorities.

The principles for publication of Results of this study will be addressed in Clinical Trial Agreements between Starpharma and the study site, and Starpharma and the subcontractors performing the study.

[Note: Results means any and all information and know how (whether patentable or not) which is discovered, invented or developed or which arises in the course of or as a result of the conduct of the Study, including any and all improvements to the products being studied.]

16. APPENDICES

16.1 Declaration of Helsinki

The Declaration of Helsinki can be found at the following link:

http://www.wma.net/e/policy/b3.htm

16.2 Toxicity Tables

The severity of AEs experienced by participants are graded in the first instance according to the Division of AIDS Female Genital Grading Table for Use in Microbicide Studies, which may be found in the Study Procedures Manual (MOP).

Those events not listed in the Female Genital Grading Table, will be graded according to the Division of AIDS Table for Grading the Severity of Adult and Pediatric Adverse Events (DAIDS AE Grading Table). The latest version can be found at the following link (and is also found in the Study Procedures Manual):

http://rcc.tech-res.com/tox_tables.htm

If events are not listed in either table, then they may be graded as per Section 8.3 of the protocol.

Definitions as found in the DAIDS tables:

Basic Self-care functions: Activities such as bathing, dressing, toileting, transfer/movement, continence, and feeding.

LLN – Lower limit of normal

Medical Intervention – Use of pharmacologic or biologic agent(s) for treatment of an AE.

N/A – Not applicable

Operative Intervention – Surgical OR other invasive mechanical procedures.

ULN – Upper limit of normal

Usual Social & Functional Activities – Adaptive tasks and desirable activities, such as going to work, shopping, cooking, use of transportation, pursuing a hobby etc.

16.3 Follow-up Evaluations

16.3.1 Clinical Evaluation upon Reporting GU Symptoms

Condition / Complaint	Study Gel Use	Evaluation	Follow up and Treatment Action
Vulval irritation, swelling, sores	Hold study gel use until evaluated (within 24 hours)	Genital examination	Follow up according to signs provided in Table 2 below. If antibiotic treatment required patient must cease treatment, but is to continue study visits. If no significant clinical signs, restart treatment with agreement of patient at the discretion of the investigator.
Dysuria, frequency of micturition	Hold study gel use until evaluated (within 24 hours)	Urine dipstick and midstream urine for culture and antimicrobial sensitivity testing	If antibiotic treatment required, patient must cease treatment, but is to contine study visits. Reinstate study gel with agreement of patient at the discretion of the investigator if symptoms resolve within 24-48 hours, otherwise withdraw patient.
Vaginal discomfort, superficial Dyspareunia, vaginal discharge (change from normal)	Hold study gel use until evaluated (within 24 hours)	Genital and vaginal speculum examination. Bimanual pelvic exam if pelvic pain	Follow-up according to signs provided in Table 2 below. If no significant signs are observed, re-instate study gel with the permission of the patient at discretion of the investigator
Intermenstrual Bleeding /Spotting	Hold study gel use until evaluated (within 24 hours)	Genital and vaginal speculum examination	If determined to be endometrial bleeding with no other source, re-instate study gel use with the agreement of the patient at the discretion of the investigator. Re-evaluate in 24-48 hours if the participant reports the bleeding / spotting has not resolved.
SAE that is judged by the site investigator or designee to be definitely, probably, possibly, or probably not related to the study gel	For Grades 1,2, and 3 – Hold study gel use until evaluated (within 24 hours)<br><br>For Grade 4 – Permanent Discontinuation	Evaluate as according to current clinical practice at the site<br><br>Not Applicable	Provide treatment as clinically indicated. If resolved within 24-48 hours, reinstate study gel use with patient's permission at investigator's discretion. If antibiotic treatment required, patient cease treatment, but is to continue study visits.<br><br>Not Applicable

16.3.2 Follow up of findings seen on pelvic exam

Condition / complaint	Study Gel Use	Evaluation	Follow up and Treatment Action
Localized vulval erythema involving less than 33% of introital surface with intact epithelial surfaces:	Continue at clinician's discretion	External genital visual examination	If asymptomatic, re-evaluate at next regularly scheduled visit. If symptomatic, re-evaluate in 24 hours. If worsened significantly, hold study gel use, and re-evaluate in 24 hours and re-instate study gel if resolved. Otherwise, gel may be continued or permanently discontinued at the investigator's discretion.
Generalized vulval erythema or severe edema; area of more than 33% of introital surface	Hold study gel use until re-evaluated	External genital visual examination	Re-evaluate in 24 hours and reinstate study gel use if resolved.<br>If there is reoccurrence and there is no other etiology, then consider permanent discontinuation at the investigator's discretion.
Localized vaginal inflammation involving less than 33% surface area without change in usual vaginal discharge	Continue at clinician's discretion	Vaginal speculum examination	If asymptomatic, re-evaluate at next regularly scheduled visit. If symptomatic, re-evaluate in 24 hours. If worsened significantly, hold study gel use, and re-evaluate in 24 hours. If condition resolves re-instate study gel with agreement of patient at investigator's discretion. Otherwise, consider permanent discontinuation at the investigator's discretion.
Generalized vaginal inflammation involving >33% surface area and / or change in usual vaginal discharge	Hold study gel use until evaluated	Vaginal speculum examination	Assess vaginal microflora by PH, Gram stain and wet prep microscopy (culture for yeasts and bacterial pathogens according to local protocol). Treat according to findings as per the protocol if condition not associated with BV and re-evaluate in 24-48 hours. If treatment requires antibiotics or vaginal preparations patient must cease treatment, but is to contine study visits. May continue study gel use if symptoms improved during re-evaluation. If there is recurrence, then consider permanent discontinuation.
Superficial Epithelial Disruption (Abrasion/Peeling)	Hold study gel use until re-evaluated	External genital visual examination	Re-evaluate in 24 hours. If condition is significantly worse permanently discontinue study gel use. Otherwise continue study gel use with patient's permission at investigator's discretion.
Deep Epithelial Disruption (Ulceration)	Hold use of study gel until ulceration evaluated	Swab for herpes simplex culture or PCR Swab for bacteriology if	Re-evaluate in 24-48 hours and reinstate study gel use if resolved. If the ulcer has become worse or not healed, or requires antibiotic treatment,

		clinically indicated.<br>Perform syphilis serology (Herpes serology optional).<br>At clinician discretion, perform dark field microscopy for syphilis	permanently discontinue study gel and consider a biopsy. Follow-up syphilis serology will be necessary for up to 3 months thereafter.
Suspected cervicitis (findings on exam such as discharge from the cervical os)	Continue at clinician's discretion	Evaluate for N. gonorrhea and C. trichomoniasis.	Re-evaluate in 24 hours. If condition is worse, hold study gel and re-evaluate in 24 hours. If condition resolves, re-instate study gel. If condition continues, consider permanently discontinuing study gel at investigator's discretion.

16.4 References

1. International Conference on Harmonisation Guidelines E6, Good Clinical Practice, July 96, issued as CPMP/ICH/135/95/Step5, Explanatory Note and Comments issued as CPMP/768/97
2. Josey WE, Schwebke JR. The polymicrobial hypothesis of bacterial vaginosis causation: a reassessment. *Intl. J. STD AIDS* 2008;19:152-154.
3. Hillier SL, Nugent RP, Eschenbach DA et al. Association between bacterial vaginosis and preterm delivery of a low-birth-weight infant. The Vaginal Infections and Prematurity Study Group. *NEJM*. 1995;333:1737-1742.
4. Taha TE, Hoover DR, Dallabetta GA et al. Bacterial vaginosis and disturbances of vaginal flora: association with increased acquisition of HIV. *AIDS*. 1998; 12:1699-1706.
5. Bradshaw CS, Morton AN, Hocking J et al. High recurrence rates of bacterial vaginosis over the course of 12 months after oral metronidazole therapy and factors associated with recurrence. *J. Infect. Dis.* 2006; 193:1478-1486.
6. Frechet JM, Tomalia DA. Dendrimers and Other Dendritic Polymers. Chichester:Wiley, 2001.
7. Starpharma Pty Ltd. Investigators' Brochure: SPL7013 Gel (VivaGel®). Document and Version No: CIB-002-01. Release Date: June 2010
8. Witvrouw M, Fikkert V, Pluymers W, et al. Polyanionic (i.e. polysulfonate) dendrimers can inhibit the replication of human immunodeficiency virus by interfering with both virus adsorption and later steps (reverse transcriptase/integrase) in the virus replicative cycle. Mol. Pharmacol. 2000 Nov;58(5):1100-8.
9. Bernstein DI, Stanberry LR, Sacks S, et al. Evaluations of unformulated and formulated dendrimer-based microbicide candidates in mouse and guinea pig models of genital herpes. Antimicrob Agents Chemother 2003 Dec;47(12):3784-8
10. Bourne N, Stanberry LR, Kern ER, Holan G, Matthews B, Bernstein DI. Dendrimers, a new class of candidate topical microbicides with activity against herpes simplex virus infection. Antimicrob Agents Chemother 2000 Sep;44(9):2471-4.
11. Jiang YH, Emau P, Cairns JS, et al. SPL7013 gel as a topical microbicide for prevention of vaginal transmission of $SHIV_{89.6p}$ in macaques. AIDS Res Hum Retroviruses 2005 Mar;21(3):207-13
12. Abdool Karim S, Coletti A, Richardson B, Ramjee G, Hoffman I, Chirenje M, Taha T, Kapina M, Maslankowski S, Soto-Torres L. Safety and Effectiveness of Vaginal Microbicides BufferGel and 0.5% PRO 2000/5 Gel for the Prevention of HIV Infection in Women: Results of the HPTN 035 Trial. 16th CROI 2009, Oral Abstract 43LB.
13. Chisembele M, Crook A, Gafos M, Hayes R, Jentsch U, Kamali A, Lacey C, McCormack C, Ramjee G, Rees H, and the MDP Team. PRO2000 Vaginal Gel is Ineffective in Preventing HIV Infection: Results of the MDP301 Phase III Microbicide Trial. 17th CROI 2010. Paper 87LB

14. McCormack S, Chisembele M, Gilbert C, Gafos M, Hayes R, Jentsch U, Kamali A, Lacey C, Ramjee G, Rees H. Assessing the Safety of PRO2000 Vaginal Gel in the MDP301 Phase III Microbicide Trial. Microbicides 2010. Oral Abstract 26.
15. Amsel R, Totten PA, Spiegel CA, Chen KCS, Eschenbach D, Holmes KK. Nonspecific Vaginitis: Diagnostic criteria and microbial and epidemiologic associations. Am J Med. 1983;74:14-22
16. Nugent RP, Krohn MJ, Hillier SL. Reliability of diagnosing bacterial vaginosis is improved by a standardized method of Gram stain interpretation. J Clin Microbiol 1991;29:297-301
17. Atkinson MJ, Sinha A, Hass A, et al. Validation of a general measure of treatment satisfaction, the Treatment Satisfaction Questionnaire for Medication (TSQM) using a national panel study of chronic disease. Health and Quality of Life Outcomes, 2004:2:12.
